# Relationship of extracellular volume assessed on cardiac magnetic resonance and serum cardiac troponins and natriuretic peptides with heart failure outcomes

**DOI:** 10.1038/s41598-019-56213-4

**Published:** 2019-12-27

**Authors:** Eric Y. Yang, Mohammad A. Khan, Edward A. Graviss, Duc T. Nguyen, Arvind Bhimaraj, Vijay Nambi, Ron C. Hoogeveen, Christie M. Ballantyne, William A. Zoghbi, Dipan J. Shah

**Affiliations:** 1 Houston Methodist Hospital, Houston, TX USA; 2 Michael E. DeBakey Veterans Affairs Medical Center, Houston, TX USA; 3 Department of Medicine, Baylor College of Medicine, Houston, TX USA

**Keywords:** Prognostic markers, Cardiology

## Abstract

Measures of serum cardiac troponins and natriuretic peptides have become established as prognostic heart failure risk markers. In addition to detecting myocardial fibrosis through late gadolinium enhancement (LGE), extracellular volume fraction (ECV) measures by cardiac magnetic resonance (CMR) have emerged as a phenotypic imaging risk marker for incident heart failure outcomes. We sought to examine the relationship between cardiac troponins, natriuretic peptides, ECV and their associations with incident heart failure events in a CMR referral base. Mid short axis T1 maps were divided into 6 cardiac segments, each classified as LGE absent or present. Global ECV was derived from T1 maps using the area-weighted average of only LGE-absent segments. ECV was considered elevated if measured >30%, the upper 95% bounds of a reference healthy group without known cardiac disease (*n* = 28). Patients were dichotomized by presence of elevated ECV. High-sensitivity cardiac troponin T (hs-cTnT) and N-terminal B-type natriuretic peptide (NT-proBNP) were measured using serum samples acquired and stored at time of CMR scan, and patients were categorized into 3 groups for each blood marker based on recommended cutoff values. Subsequent heart failure admission and any death were ascertained. Relationships with ECV, hs-cTnT, and NT-proBNP were examined separately and as a composite with Cox proportional hazard models. Of 1,604 serial patients referred for a clinical CMR with myocardial T1 maps, 331 were eligible after exclusions and had blood available and were followed over a median 25.0 [interquartile range 21.8, 31.7] months. After adjustments for age (mean 57.3 [standard deviation (SD) 15.1 years), gender (61% male), and ethnicity (12.7% black), elevated ECV remained a predictor of a first composite heart failure outcome for patients with high levels of hs-cTnT (≥14 ng/L; hazard ratio [HR] 2.42 [95% confidence interval (CI) 1.17, 5.03]; p = 0.02) and NT-proBNP (≥300 pg/mL; HR 2.28 [95% CI 1.24, 4.29]; p = 0.01). Similar trends were seen for lower category levels of blood markers, but did not persist with minimal covariate adjustments. Elevated measures of ECV by CMR are associated with incident heart failure outcomes in patients with high hs-cTnT and NT-proBNP levels. This imaging marker may have a role for additional heart failure risk stratification.

## Introduction

Non-invasive measures of extracellular volume fraction (ECV) by cardiac magnetic resonance techniques (CMR) have been histologically validated as surrogates of the myocardial interstitial volume, have been shown to be altered in various heart diseases, and have emerged as potential imaging risk markers for subsequent heart failure events^1–5^. Elevations in measures of natriuretic peptides and cardiac troponins have previously been established as markers of poor heart failure prognosis not only in patients with established heart failure (i.e., a secondary prevention population) but also in patients without established disease (i.e., a primary prevention population at risk for heart failure)^6,7^. The detection of elevated natriuretic peptides through B-type natriuretic peptide (BNP) and its propeptide N-type proBNP (NT-proBNP) has entered clinical use in diagnosing and guiding management of patients with or at risk for heart failure, and for risk prognostication in patients with chronic heart failure per guideline recommendations^6,8^. Assays for measuring cardiac troponins have improved in the last several years with newer commercial assays that enable lower limits of measurements using high sensitivity techniques.

Epidemiologic studies have established a strong association between troponin levels detected at lower levels with subsequent adverse cardiovascular events in various populations^9^. Limited data exist on the association between such imaging and blood markers and would be essential to formulating future research strategies and clinical recommendations for improving heart failure risk stratification using such biomarker combinations. In this study, we sought to examine the relationships between ECV, NT-proBNP, high-sensitivity cardiac troponin T (hs-cTnT) and incident heart failure outcomes in a population referred for CMR.

## Methods

### Study population

The study was approved by the local institutional review board of the Houston Methodist Research Institute and conducted in accordance with Good Clinical Practice guidelines^10^. Written informed consent was obtained from all participants prior to enrollment. Subjects were serially recruited from patients referred to the cardiovascular magnetic resonance (CMR) laboratories of Houston Methodist Hospital, a tertiary center for routine CMR imaging from June 2011 through January 2015, as part of an ongoing prospective cohort study designed to examine the relationship of myocardial extracellular volume fraction (ECV) with heart failure events. All patients were approached during this time period by the CMR staff on arrival to the waiting area, at which point the study was discussed and the consent form was provided. When patients were brought the holding area for safety screening and final preparations before the CMR scan, they were given the opportunity to ask questions about the study and to choose to participate.

Indications for referral are included in Supplement Table 1, and patients could have multiple indications (i.e., not mutually exclusive indications) for a CMR study. Patients were included if aged ≥18 years, able to receive intravenous gadolinium-based contrast agents (i.e., no prior allergy to gadolinium agents, estimated glomerular filtration rate ≥30 mL/min/1.73 m^2^), had no contraindications to CMR, and completed the full imaging protocol which included, cine-CMR, late gadolinium enhancement (LGE), and T1 mapping procedure (i.e., pre- and post-contrast imaging, hematocrit levels available within ≤90 days of CMR scan because hematocrit is used in the derivation of ECV). Exclusion criteria included any missing relevant demographic data, refusing or missing blood samples, technical issues with imaging, and any infiltrative cardiomyopathy or any cardiac tumors by clinical history or detected on CMR. Three hundred thirty one participants were selected for analyses after application of inclusion and exclusion criteria (please see Supplemental Fig. 1). Healthy volunteers without any known cardiovascular diseases or risk factors were also recruited to determine the distribution of ECV in a reference control group (Supplemental Methods).

### Outcomes

Follow-up of subjects was conducted through structured phone interviews with the participants, review of the available electronic health records (EHR), or contact with the referring clinic through December 31, 2016. The primary outcome of the first major heart failure event was a composite of heart failure hospitalization and death from any cause. Individual outcome types were examined separately as secondary outcomes. As established in the 2014 American College of Cardiology/American Heart Association (AHA) Cardiovascular Endpoints Data Standards, heart failure hospitalization events were defined as any hospitalization with a primary diagnosis of heart failure where the patient had a length of stay of at least 24 hours, symptoms or objective evidence of new or worsening heart failure, and initiation or intensification of heart failure therapies^11^. Deaths were ascertained from EHR review and/or Social Security Death Index queries. Events were adjudicated by a committee consisting of three board-certified cardiologists.

### CMR imaging

Patients underwent CMR scans on either a 1.5-Tesla or a 3.0-Tesla clinical scanner (Siemens Avanto or Verio, respectively; Siemens, Erlangen, Germany) with phased-array receiver coil systems. Imaging protocols consisted at a minimum of an electrocardiography (ECG)-gated cine section and LGE imaging as previously described, and each of these protocol sections was followed with modified Look-Locker inversion recovery (MOLLI) sequences for T1 mapping just prior to and ~15 minutes after contrast administration^12–17^.

#### Volumes & function

Briefly, cardiac cines were acquired with a steady-state free precession sequence (SSFP) over 25–30 cardiac phases with in-plane spatial resolutions of 1.7 to 2.0 mm by 1.4 to 1.6 mm using sequential short-axis stacks (i.e., ventricular base to apex) with 10-mm increments (6-mm thickness, 4-mm gap) and standard cardiac long-axis views (i.e., left ventricular [LV] based 3-, 4-, and 2-chamber views). Cardiac chamber parameters were measured by level III trained CMR readers and indexed for body surface area^18^.

#### Late gadolinium enhancement

LGE images were acquired over slice positions matched to cines about 10–15 minutes following intravenous gadolinium-based contrast administration (gadopentetate dimeglumine, gadoterate meglumine; 0.15 mmol/kg) with in-plane spatial resolutions of 1.8 mm by 1.3 mm and slice thicknesses of 6–7 mm with 3–4 mm gap. LGE images were obtained using inversion-recovery gradient echo sequences with inversion times set to null myocardial tissue signal (inversion time [TI] 250–350 msec)^19,20^.

The presence and extent of myocardial LGE, considered synonymous with replacement fibrosis or scar tissue, was assessed using the AHA 17-segment model by level III trained CMR readers^18,21^. For each myocardial segment, the extent of regional LGE was scored according to the spatial extent of LGE within each segment (0 = no LGE; 1 = 1%–25% LGE; 2 = 26%–50% LGE; 3 = 51%–75% LGE; and 4 = 76%–100% LGE). The total scar burden for the entire left ventricle was expressed as a percentage of LV myocardial volume and derived by averaging the score for all 17 segments.

#### T1 Mapping & ECV

An ECG-gated MOLLI sequence with SSFP image readout with motion correction was performed at a representative mid short-axis view of the left ventricle at two distinct time points within a CMR scan: once following cine imaging but prior to contrast administration (pre-contrast), and once ~15 minutes following LGE imaging (post-contrast). The mid short-axis was chosen due to concerns with partial volume due to through-plane cardiac motion near the base and near the apex. Technical details of the MOLLI sequence setup can be found in the Supplemental Material.

A single reader – blinded to the clinical history, other CMR images, and clinical outcomes – post-processed all cases in randomized order and abstracted the T1 values for ECV calculations. Intra- and inter-reader reproducibility of ECV measures were separately assessed in a selected sub-group, which was re-randomized for each reader (Supplemental Material). ECV was assessed for the 6 myocardial segments of the mid short axis using post-processing image analysis tools (cvi^42^ software, Circle Cardiovascular Imaging, Calgary, Canada; Supplemental Material). The LGE image corresponding to the T1 maps was reviewed for LGE presence. Myocardial segments with LGE were classified as coronary artery disease (CAD) or non-CAD type LGE for subsequent exclusion from ECV calculations.

For each of the 6 myocardial segments, myocardial ECV was derived using pre- and post-contrast T1 values of myocardium and blood pool as previously described in other works^22,23^. Briefly, ECV was calculated as (1 − Hct) × (ΔR1_myocardium_/ΔR1_blood_), where Hct is the hematocrit level and ΔR1 represents the change in T1 relaxivity (R1 = 1/T1) before and after gadolinium-based contrast administration. Myocardial segments with any artifacts or LGE were excluded from the ECV calculation used for the main analyses. Segments with LGE were excluded because LGE has been determined to increase ECV and to be independently associated with adverse cardiovascular outcomes. A global scar-free ECV was then calculated, average-weighted for the size of each usable myocardial segment.

### Blood storage & assay protocols

Blood was drawn in ethylenediamine tetraacetic acid tubes, mixed and placed on ice, or into serum separator tubes, which were allowed to stand for 30–45 minutes at room temperature. These tubes were sent within the hour to the onsite research core laboratory for further processing. Tubes were centrifuged at 3000 rpm for 15 minutes at ambient temperature. The plasma or serum was transferred from the tubes with a sterile pipette into three 1 mL aliquots for storage. Samples were frozen and stored at − 80 °C.

A 1 mL aliquot for each eligible study participant with available blood samples was transferred to the Atherosclerosis Clinical Research Laboratory (ACRL) at the Baylor College of Medicine and thawed to ambient temperature prior to use. Plasma levels of cardiac troponin T were measured using a high-sensitivity assay (Elecsys-2010 Troponin T hs STAT, Roche Diagnostics) on a Cobas e411 automated analyzer. The hs-cTnT assay has a inter-assay coefficient of variation (CV) of 5.2% at a mean hs-cTnT concentration of 13.5 ng/L. Plasma N-terminal pro-B-type natriuretic peptide (NT-proBNP levels) were measured by electrochemoluninescence immunoassay (ECLIA) (Elecsys proBNP, Roche Diagnostics) on a Cobas e411 automated analyzer on the same sample set. The NT-proBNP assay has an inter-assay coefficient of variation (CV) of <4.6.

### Statistical methods

All statistical analyses were conducted using Stata 15.1 (StataCorp, LLC, College Station, TX). From the 28 healthy volunteers, the upper 95% cutoff or mean + 2 standard deviations (SD) of ECV was determined to be 30.2% on the 1.5-Tesla scanner and 31.1% on the 3.0-Tesla scanner. The main cohort was dichotomized into low (≤mean + 2 SD) vs. high (>mean + 2 SD) ECV categories based on ECV values and scanner type. It was also separated into three categories of hs-cTnT (<5 ng/L, 5 to 13 ng/L, and ≥14 ng/L) and into three categories of NT-proBNP (<100 pg/mL, 100 to 299 pg/mL, and ≥300 pg/mL). We elected to present results using categories for the imaging and blood biomarkers because the few number of heart failure events limited our power to examine their associations with events as continuous variables. The power calculation for our study sample was calculated for the difference in mortality, heart failure hospitalization and composite of mortality or heart failure hospitalization between the low and high ECV groups. The power calculated was based on the 2-sided chi-square test for two proportions. Analyses of the markers as continuous variables are included in the supplemental.

Baseline characteristics and CMR parameters were described. All statistical tests were two-sided with significance set at p < 0.05. Nominal variables were compared among ECV and scar groups using Chi-squared or Fisher’s exact testing as appropriate. Normality of continuous variables was assessed using Shapiro-Wilk testing. Because continuous variables were predominantly non-normally distributed, non-parametric comparison among ECV and scar groups were conducted for all continuous variables using Kruskal Wallis testing. Correlations between continuous ECV measures and blood biomarkers were assessed using Spearman’s rank correlation coefficients. Associations between blood biomarkers and binary ECV categories were further assessed using multivariable logistic regression. We examined a parsimonious logistic regression model, developed using Bayesian model averaging to select out only significant predictors of elevated ECV^24,25^.

Time to first composite event of either heart failure hospitalization or any death event were depicted using Kaplan-Meier survival curves. Differences between groups was compared by the log-rank test. Potential risk factors associated with the composite event were determined by and univariable and multivariable Cox proportional hazard risk models examining ECV as a binary variable (normal vs. elevated, based on cutoffs derived from the healthy volunteers). Based on a previously validated heart failure prediction model from the Atherosclerosis Risk in Communities Study (ARIC)^26^, we employed a base multivariable model with stepwise addition of covariate clusters (imaging and blood markers) to assess whether scar-free ECV categories contributed to the model for prediction of incident heart failure events. This multivariable model included age, sex, black ethnicity, current and former smoking status, heart rate, body mass index, history of hypertension, history of diabetes, and history of prior myocardial infarction.

Harrell’s C-statistic was used to compare the diagnostic performance of the predictive models. The incremental benefit of scar-free ECV categories when added back to the multivariate models was evaluated using the post-hoc linear combinations of estimators^27^.

## Results

Overall, the cohort had a median age of 59.7 (interquartile range 47.4 to 67.8) years with a male predominance (Tables 1 and 2). The top three most frequent indications for CMR study referral were cardiomyopathy (31.7%), valve (31.1%), and viability (11.8%) assessment (Supplemental Table 1). Participants with higher levels of hs-cTnT tended to be older and male and to have a history of hypertension, diabetes, and prior myocardial infarction (Table 1). They also had a greater prevalence for use of aspirin, thienopyridine, renin-angiotensin-aldosterone system inhibitors, beta-blockers, diuretics, amiodarone, insulin, oral hypoglycemic medications, and 3-hydroxy-3-methylglutaryl-coenzyme A reductase inhibitors (aka statins). Participants with higher hs-cTnT levels also tended to have larger LV end systolic volumes, stroke volumes, and myocardial mass; smaller right ventricular chamber and stroke volumes; and lower biventricular ejection fractions (Table 3). Similar trends in baseline characteristics was observed for participants with higher levels of NT-proBNP (Tables 2 and 4). In addition, patients with higher levels of NT-proBNP tended to have lower diastolic blood pressures and tended to use aldosterone antagonists and nitrate medications more frequently.

**Table 1:** Baseline characteristics and categorization by high sensitivity cardiac troponin T levels.

	All	High-sensitivity troponin (ng/L)			
		<0.005	0.005–0.013	≥0.014	Overall p-value
	(N = 331)	(*n* = 120)	(*n* = 98)	(*n* = 113)	
Age (yrs), median (IQR)	59.7 (47.4, 67.8)	51.8 (38.1, 60.8)	58.3 (49.2, 67.4)	67.0 (60.2, 73.5)	<0.001
Male	202 (61.0%)	58 (48.3%)	63 (64.3%)	81 (71.7%)	<0.001
Race					0.004
White	251 (75.8%)	101 (84.2%)	64 (65.3%)	86 (76.1%)	
Black	42 (12.7%)	6 (5.0%)	22 (22.4%)	14 (12.4%)	
Asian	6 (1.8%)	4 (3.3%)	1 (1.0%)	1 (0.9%)	
Other	32 (9.7%)	9 (7.5%)	11 (11.2%)	12 (10.6%)	
Black					<0.001
No	289 (87.3%)	114 (95.0%)	76 (77.6%)	99 (87.6%)	
Yes	42 (12.7%)	6 (5.0%)	22 (22.4%)	14 (12.4%)	
History of hypertension	197 (59.5%)	47 (39.2%)	63 (64.3%)	87 (77.0%)	<0.001
History of dyslipidemia	155 (46.8%)	29 (24.2%)	53 (54.1%)	73 (64.6%)	<0.001
History of myocardial infarction	42 (12.7%)	3 (2.5%)	16 (16.3%)	23 (20.4%)	<0.001
History of diabetes	60 (18.2%)	9 (7.6%)	10 (10.2%)	41 (36.3%)	<0.001
Smoking					
Current	28 (8.6%)	9 (7.5%)	8 (8.2%)	11 (10.0%)	0.13
Former (>1 yr)	98 (30.0%)	28 (23.3%)	28 (28.9%)	42 (38.2%)	
Systolic blood pressure (mm Hg), median (IQR)	127.0 (116.0, 138.0)	126.0 (118.0, 136.0)	129.0 (117.0, 139.0)	125.0 (113.0, 138.0)	0.47
Diastolic blood pressure (mm Hg), median (IQR)	75.0 (66.0, 84.0)	75.5 (68.5, 84.0)	75.5 (68.0, 84.0)	72.0 (64.0, 81.0)	0.08
Heart Rate (bpm), median (IQR)	70.0 (61.0, 81.0)	69.5 (61.0, 81.0)	68.0 (60.0, 80.0)	73.0 (65.0, 82.0)	0.06
Body mass index (kg/sq. m), median (IQR)	28.0 (24.7, 32.4)	26.5 (23.5, 31.2)	28.7 (25.2, 34.2)	28.3 (26.3, 32.4)	0.01
**Medication usage**					
Aspirin	114 (34.5%)	27 (22.5%)	31 (32.0%)	56 (49.6%)	<0.001
Thienopyridine	32 (9.7%)	6 (5.0%)	9 (9.2%)	17 (15.0%)	0.03
Warfarin	59 (17.8%)	15 (12.5%)	18 (18.4%)	26 (23.0%)	0.11
ACE inhibitor	96 (29.0%)	21 (17.5%)	33 (33.7%)	42 (37.2%)	0.002
Angiotensin II receptor blocker	56 (16.9%)	11 (9.2%)	14 (14.3%)	31 (27.4%)	<0.001
Spironolactone	33 (10.0%)	6 (5.0%)	11 (11.2%)	16 (14.2%)	0.06
Diuretics	113 (34.1%)	19 (15.8%)	28 (28.6%)	66 (58.4%)	<0.001
Insulin	19 (5.7%)	1 (0.8%)	5 (5.1%)	13 (11.5%)	0.002
Oral hypoglycemic medication	39 (11.8%)	6 (5.0%)	9 (9.2%)	24 (21.2%)	<0.001
Statin	144 (43.5%)	25 (20.8%)	49 (50.0%)	70 (61.9%)	<0.001
Hormone replacement therapy	26 (7.9%)	12 (10.0%)	7 (7.1%)	7 (6.2%)	0.53
β-blockers	183 (55.3%)	55 (45.8%)	53 (54.1%)	75 (66.4%)	0.01
Calcium channel blockers	41 (12.4%)	10 (8.3%)	14 (14.3%)	17 (15.0%)	0.24
Digoxin	31 (9.4%)	7 (5.8%)	9 (9.2%)	15 (13.3%)	0.15
Amiodarone	22 (6.6%)	3 (2.5%)	4 (4.1%)	15 (13.3%)	0.002
Nitrates	27 (8.2%)	4 (3.3%)	10 (10.2%)	13 (11.5%)	0.051

IQR, interquartile range; ACE = angiotension converting enzyme.

**Table 2:** Baseline characteristics and categorization by N-terminal pro B-type natriuretic peptide levels.

	All	NT-proBNP (pg/mL)			
		<100	100–299	≥300	Overall p-value
	(N = 331)	(*n* = 78)	(*n* = 95)	(*n* = 158)	
Age (yrs), median (IQR)	59.7 (47.4, 67.8)	45.9 (35.2, 58.2)	57.6 (46.9, 65.8)	65.0 (57.1, 72.5)	<0.001
Male	202 (61.0%)	52 (66.7%)	56 (58.9%)	94 (59.5%)	0.50
Race					0.36
White	251 (75.8%)	58 (74.4%)	75 (78.9%)	118 (74.7%)	
Black	42 (12.7%)	10 (12.8%)	10 (10.5%)	22 (13.9%)	
Asian	6 (1.8%)	1 (1.3%)	4 (4.2%)	1 (0.6%)	
Other	32 (9.7%)	9 (11.5%)	6 (6.3%)	17 (10.8%)	
Black					0.73
No	289 (87.3%)	68 (87.2%)	85 (89.5%)	136 (86.1%)	
Yes	42 (12.7%)	10 (12.8%)	10 (10.5%)	22 (13.9%)	
History of hypertension	197 (59.5%)	28 (35.9%)	53 (55.8%)	116 (73.4%)	<0.001
History of dyslipidemia	155 (46.8%)	26 (33.3%)	43 (45.3%)	86 (54.4%)	0.01
History of myocardial infarction	42 (12.7%)	2 (2.6%)	9 (9.5%)	31 (19.6%)	<0.001
History of diabetes	60 (18.2%)	7 (9.0%)	11 (11.6%)	42 (26.8%)	<0.001
Smoking					0.21
Current	28 (8.6%)	6 (7.7%)	5 (5.3%)	17 (11.0%)	
Former (>1 yr)	98 (30.0%)	17 (21.8%)	29 (30.5%)	52 (33.8%)	
Systolic blood pressure (mm Hg), median (IQR)	127.0 (116.0, 138.0)	127.5 (118.0, 136.0)	126.0 (117.0, 138.0)	126.5 (113.0, 138.0)	0.63
Diastolic blood pressure (mm Hg), median (IQR)	75.0 (66.0, 84.0)	77.5 (70.0, 86.0)	75.0 (66.0, 84.0)	73.0 (65.0, 82.0)	0.04
Heart Rate (bpm), median (IQR)	70.0 (61.0, 81.0)	68.0 (60.0, 77.0)	68.0 (61.0, 80.0)	73.5 (64.0, 83.0)	0.01
Body mass index (kg/sq. m), median (IQR)	28.0 (24.7, 32.4)	27.2 (24.5, 30.4)	27.8 (24.6, 33.1)	28.2 (25.1, 32.8)	0.20
**Medication usage**					
Aspirin	114 (34.5%)	20 (26.0%)	25 (26.3%)	69 (43.7%)	0.004
Thienopyridine	32 (9.7%)	1 (1.3%)	12 (12.6%)	19 (12.0%)	0.02
Warfarin	59 (17.8%)	5 (6.4%)	16 (16.8%)	38 (24.1%)	0.004
ACE inhibitor	96 (29.0%)	13 (16.7%)	28 (29.5%)	55 (34.8%)	0.02
Angiotensin II receptor blocker	56 (16.9%)	5 (6.4%)	15 (15.8%)	36 (22.8%)	0.01
Spironolactone	33 (10.0%)	2 (2.6%)	6 (6.3%)	25 (15.8%)	0.002
Diuretics	113 (34.1%)	8 (10.3%)	22 (23.2%)	83 (52.5%)	<0.001
Insulin	19 (5.7%)	2 (2.6%)	5 (5.3%)	12 (7.6%)	0.29
Oral hypoglycemic medication	39 (11.8%)	4 (5.1%)	9 (9.5%)	26 (16.5%)	0.03
Statin	144 (43.5%)	24 (30.8%)	36 (37.9%)	84 (53.2%)	0.002
Hormone replacement therapy	26 (7.9%)	4 (5.1%)	9 (9.5%)	13 (8.2%)	0.56
β-blockers	183 (55.3%)	30 (38.5%)	41 (43.2%)	112 (70.9%)	<0.001
Calcium channel blockers	41 (12.4%)	4 (5.1%)	10 (10.5%)	27 (17.1%)	0.03
Digoxin	31 (9.4%)	1 (1.3%)	7 (7.4%)	23 (14.6%)	0.003
Amiodarone	22 (6.6%)	0 (0.0%)	4 (4.2%)	18 (11.4%)	0.002
Nitrates	27 (8.2%)	2 (2.6%)	4 (4.2%)	21 (13.3%)	0.01

IQR, interquartile range; ACE = angiotensin converting enzyme.

**Table 3:** Cardiac magnetic resonance characteristics and categorization by high-sensitivity cardiac troponin T levels.

	All	High-sensitivity troponin (ng/mL)			
		<0.005	0.005–0.013	≥0.014	Overall p-value
	(N = 331)	(*n* = 120)	(*n* = 98)	(*n* = 113)	
**Cardiac magnetic resonance characteristics**					
Left Ventricle					
EDVi (mL/sq. m), median (IQR)	71.9 (57.4, 95.8)	69.6 (57.0, 81.3)	70.4 (57.4, 98.6)	76.5 (59.2, 114.3)	0.04
ESVi (mL/sq. m), median (IQR)	25.7 (17.4, 43.4)	24.0 (17.0, 31.4)	25.6 (16.6, 53.8)	31.8 (19.0, 67.6)	<0.001
SVi (mL/sq. m), median (IQR)	42.2 (34.7, 49.3)	45.3 (38.3, 51.7)	40.3 (33.6, 48.1)	38.2 (33.5, 47.2)	<0.001
EF (%), median (IQR)	64.0 (48.0, 72.0)	66.0 (61.0, 73.0)	63.0 (43.0, 72.0)	57.0 (34.0, 69.0)	<0.001
MMi (gm/sq. m), median (IQR)	70.3 (56.1, 91.3)	61.1 (51.0, 74.5)	74.0 (58.6, 95.4)	78.6 (65.4, 103.3)	<0.001
Right Ventricle					
EDVi (mL/sq. m), median (IQR)	68.4 (56.6, 84.8)	71.9 (60.9, 86.8)	68.0 (55.8, 84.8)	64.4 (53.2, 79.5)	0.04
ESVi (mL/sq. m), median (IQR)	30.6 (24.0, 40.1)	30.5 (25.2, 39.8)	28.9 (23.0, 39.9)	31.6 (24.0, 40.6)	0.69
SVi (mL/sq. m), median (IQR)	37.2 (28.9, 45.2)	40.9 (34.6, 50.0)	36.0 (28.5, 44.8)	32.1 (25.6, 39.7)	<0.001
EF (%), median (IQR)	55.0 (49.0, 61.0)	56.5 (52.0, 61.0)	55.5 (48.0, 63.0)	51.0 (45.5, 59.0)	<0.001
**Extracellular volume (ECV) and scar**					
ECV (%), median (IQR)	28.0 (26.0, 32.0)	28 (26, 30)	28 (26, 32)	29 (28, 33)	<0.001
ECV (%)					<0.001
Normal	229 (69.2%)	99 (82.5%)	66 (67.3%)	64 (56.6%)	
Elevated	102 (30.8%)	21 (17.5%)	32 (32.7%)	49 (43.4%)	
Scar burden (% myocardium), median (IQR)	0.0 (0.0, 3.0)	0.0 (0.0, 0.0)	0.0 (0.0, 4.0)	2.0 (0.0, 7.0)	<0.001
Any scar presence					<0.001
No	206 (62.2%)	107 (89.2%)	59 (60.2%)	40 (35.4%)	
Yes	125 (37.8%)	13 (10.8%)	39 (39.8%)	73 (64.6%)	
CAD scar presence					<0.001
No	282 (85.2%)	118 (98.3%)	82 (83.7%)	82 (72.6%)	
Yes	49 (14.8%)	2 (1.7%)	16 (16.3%)	31 (27.4%)	

IQR, interquartile range; EDVi, end-diastolic volume index; ESVi, end-systolic volume index; MMi, myocardial mass index; SVi, stroke volume index; EF, ejection fraction; ECV, extracellular volume; CAD, coronary artery disease.

**Table 4:** Cardiac magnetic resonance characteristics and categorization by N-terminal pro B-type natriuretic peptide (NT-proBNP) levels.

	All	NT-proBNP (pg/mL)			
		<100	100–299	≥300	Overall p-value
	(N = 331)	(*n* = 78)	(*n* = 95)	(*n* = 158)	
**Cardiac magnetic resonance characteristics**					
Left Ventricle					
EDVi (mL/sq. m), median (IQR)	71.9 (57.4, 95.8)	71.2 (57.4, 78.4)	70.2 (57.9, 88.8)	75.2 (57.3, 113.1)	0.06
ESVi (mL/sq. m), median (IQR)	25.7 (17.4, 43.4)	22.2 (16.5, 27.4)	24.9 (17.4, 37.1)	31.4 (18.4, 68.7)	<0.001
SVi (mL/sq. m), median (IQR)	42.2 (34.7, 49.3)	44.5 (40.0, 50.1)	44.7 (37.8, 53.1)	37.6 (30.4, 46.4)	<0.001
EF (%), median (IQR)	64.0 (48.0, 72.0)	68.0 (61.0, 74.0)	66.0 (56.0, 73.0)	56.0 (35.0, 69.0)	<0.001
MMi (gm/sq. m), median (IQR)	70.3 (56.1, 91.3)	62.9 (54.0, 75.9)	65.8 (54.1, 89.3)	76.3 (61.6, 99.1)	<0.001
Right Ventricle					
EDVi (mL/sq. m), median (IQR)	68.4 (56.6, 84.8)	76.9 (65.5, 86.9)	69.8 (57.8, 82.2)	63.5 (52.6, 80.9)	<0.001
ESVi (mL/sq. m), median (IQR)	30.6 (24.0, 40.1)	33.4 (27.4, 40.4)	29.5 (23.3, 37.6)	29.9 (23.0, 40.7)	0.21
SVi (mL/sq. m), median (IQR)	37.2 (28.9, 45.2)	42.3 (37.2, 47.9)	38.5 (32.4, 48.4)	31.1 (25.7, 40.2)	<0.001
EF (%), median (IQR)	55.0 (49.0, 61.0)	56.0 (52.0, 60.0)	57.0 (51.0, 62.0)	52.0 (46.0, 60.0)	0.001
**Extracellular volume (ECV) and scar**					
ECV (%), median (IQR)	28.0 (26.0, 32.0)	26 (25, 29)	28 (26, 31)	30 (27, 33)	<0.001
ECV (%)					<0.001
Normal	229 (69.2%)	69 (88.5%)	71 (74.7%)	89 (56.3%)	
Elevated	102 (30.8%)	9 (11.5%)	24 (25.3%)	69 (43.7%)	
Scar burden (% myocardium), median (IQR)	0.0 (0.0, 3.0)	0.0 (0.0, 0.0)	0.0 (0.0, 2.0)	2.0 (0.0, 6.0)	<0.001
Any scar presence					<0.001
No	206 (62.2%)	74 (94.9%)	65 (68.4%)	67 (42.4%)	
Yes	125 (37.8%)	4 (5.1%)	30 (31.6%)	91 (57.6%)	
CAD scar presence					<0.001
No	282 (85.2%)	77 (98.7%)	82 (86.3%)	123 (77.8%)	
Yes	49 (14.8%)	1 (1.3%)	13 (13.7%)	35 (22.2%)	

IQR, interquartile range; EDVi, end-diastolic volume index; ESVi, end-systolic volume index; MMi, myocardial mass index; SVi, stroke volume index; EF, ejection fraction; ECV, extracellular volume; CAD, coronary artery disease.

Elevated scar-free ECV measures and scar presence were also more prevalent with elevated levels of blood biomarkers. Both blood biomarkers were modestly, positively correlated with elevated ECV (NT-proBNP ρ = 0.34; hs-cTnT ρ = 0.26) and scar presence (NT-proBNP ρ = 0.23; hs-cTnT ρ = 0.22) (all p < 0.001; Supplemental Table 2). Through Bayesian model averaging, age, sex, black ethnicity, history of diabetes, use of diuretics, and use of statin medications were selected for inclusion into a multivariable logistic regression model for the tendency to have elevated ECV or scar presence. In unadjusted models, increasing categories of NT-proBNP and hs-cTnT were associated with elevated ECV and scar presence (Tables 5 and 6). After adjustments for selected covariates as described, this association was maintained for elevated ECV (highest vs. lowest categories of hs-cTnT odds ratio [OR] 2.43 [95% confidence interval (CI) 1.01, 5.84], p = 0.047; highest vs. lowest categories of NT-proBNP OR 3.32 [95% CI 1.35, 8.12], p = 0.01) and even more so for scar presence (highest vs. lowest categories of hs-cTnT OR 6.26 [95% CI 2.56, 15.32], p < 0.001; highest vs. lowest categories of NT-proBNP OR 16.35 [95% CI 5.00, 53.49], p < 0.001) (Table 6). We have also analyzed the data using the continuous ECV in the multivariable linear regression models as a continuous dependent variable (for each 5% increase) with biomarkers as both continuous and categorical covariables. A significant association was found between decreased ECV and elevated biomarkers when the biomarkers were included in the models as binary covariate (elevated versus normal), but not with continuous biomarkers (Supplemental Table 3, Supplemental Fig. 2).

**Table 5:** Logistic regression analysis of the association of NT-proBNP and high-sensitivity troponin with elevated ECV and scar presence.

	Elevated ECV		Any scar (+)	
	OR (95% CI)	p-value	OR (95% CI)	p-value
**Continuous NT-proBNP and high-sensitivity troponin**				
**Unadjusted OR**				
NT-proBNP (pg/mL)	1.0002 (1.0001, 1.0004)	0.004	1.0001 (0.999, 1.0002)	0.08
High-sensitivity troponin (ng/mL)*1000	1.001 (0.999, 1.004)	0.35	1.04 (1.02, 1.06)	<0.001
**Adjusted OR**				
Adjusted for age and gender only (N = 331)				
NT-proBNP (pg/mL)	1.0002 (1.00, 1.0003)	0.04	1.00 (1.00, 1.00)	0.74
High-sensitivity troponin (ng/mL)*1000	1.00 (0.998, 1.00)	0.58	1.02 (1.01, 1.04)	0.01
Adjusted in the complete multiple logistic regression model (N = 330)*				
NT-proBNP (pg/mL)	1.00 (0.99, 1.00)	0.49	1.00 (1.00, 1.00)	0.55
High-sensitivity troponin (ng/mL)*1000	1.00 (0.998, 1.01)	0.42	1.02 (1.00, 1.03)	0.04
**Binary NT-proBNP and high-sensitivity troponin**				
**Unadjusted OR**				
Elevated NT-proBNP (≥100 pg/mL)	4.46 (2.13, 9.34)	<0.001	16.96 (6.02, 47.78)	<0.001
Elevated high-sensitivity troponin (≥0.005 ng/mL)	2.94 (1.70, 5.07)	<0.001	9.31 (4.93, 17.59)	<0.001
**Adjusted OR**				
Adjusted for age and gender only (N = 331)				
Elevated NT-proBNP (≥100 pg/mL)	3.12 (1.42, 6.85)	0.01	14.85 (4.93, 44.72)	<0.001
Elevated high-sensitivity troponin (≥0.005 ng/mL)	2.80 (1.48, 5.28)	0.002	6.66 (3.28, 13.50)	<0.001
Adjusted in the complete multiple logistic regression model (N = 330)*				
Elevated NT-proBNP (≥100 pg/mL)	2.83 (1.24, 6.48)	0.01	14.66 (4.71, 45.62)	<0.001
Elevated high-sensitivity troponin (≥0.005 ng/mL)	2.55 (1.26, 5.17)	0.01	5.80 (2.74, 12.26)	<0.001

ECV, Extracellular volume; OR, odds ratio; NT-proBNP, N-terminal pro B-type natriuretic peptide.

*Multiple logistic regression model includes NT-proBNP, high-sensitivity troponin, age (years), gender, black (versus non-black), history of diabetes, treatment with diuretics, treatment with statin.

**Table 6:** Association between ECV or any scar with elevated NT-proBNP or high-sensitivity troponin, multiple logistic regression.

	Elevated ECV			Any scar (+)		
	Adjusted OR	p-value	AUC	Adjusted OR	p-value	AUC
	(95% CI)			(95% CI)		
**Adjusted for age and gender only (N = 331)**			**0.72**			**0.83**
NT-proBNP level (pg/mL)						
<100	Ref			Ref		
100 to 299	2.33 (0.98, 5.50)	0.06		10.38 (3.26, 33.05)	<0.001	
≥300	3.74 (1.58, 8.81)	0.003		16.90 (5.32, 53.69)	<0.001	
High-sensitivity troponin level (ng/mL)						
<0.005	Ref			Ref		
0.005 to 0.013	2.23 (1.11, 4.49)	0.03		4.38 (2.02, 9.49)	<0.001	
≥0.014	2.72 (1.23, 6.01)	0.01		7.73 (3.31, 18.07)	<0.001	
Age (yrs)	1.00 (0.98, 1.02)	0.89		0.99 (0.97, 1.01)	0.46	
Male gender	0.45 (0.26, 0.76)	0.003		2.10 (1.19, 3.73)	0.01	
**Adjusted in the complete multiple logistic regression model (N = 330)**			**0.78**			**0.85**
NT-proBNP level (pg/mL)						
<100	Ref			Ref		
100 to 299	2.35 (0.96, 5.76)	0.06		11.63 (3.53, 38.31)	<0.001	
≥300	3.32 (1.35, 8.12)	0.01		16.35 (5.00, 53.49)	<0.001	
High-sensitivity troponin level (ng/mL)						
<0.005	Ref			Ref		
0.005 to 0.013	2.24 (1.04, 4.82)	0.04		4.46 (1.97, 10.07)	<0.001	
≥0.014	2.43 (1.01, 5.84)	0.047		6.26 (2.56, 15.32)	<0.001	
Age (yrs)	1.01 (0.99, 1.03)	0.42		0.98 (0.96, 1.01)	0.20	
Male gender	0.48 (0.27, 0.85)	0.01		2.07 (1.14, 3.73)	0.02	
Black	2.79 (1.28, 6.08)	0.01		1.20 (0.53, 2.73)	0.66	
History of diabetes	2.05 (1.05, 4.00)	0.04		1.95 (0.95, 3.99)	0.07	
Treatment with diuretics	1.80 (1.01, 3.20)	0.046		1.67 (0.92, 3.02)	0.09	
Treatment with statin	0.41 (0.22, 0.74)	0.003		1.63 (0.91, 2.92)	0.10	

ECV, extracellular volume; NT-proBNP, N-terminal pro B-type natriuretic peptide; OR, odds ratio; AUC, Area under the receiver operating characteristic curve (ROC).

**Figure 1:**
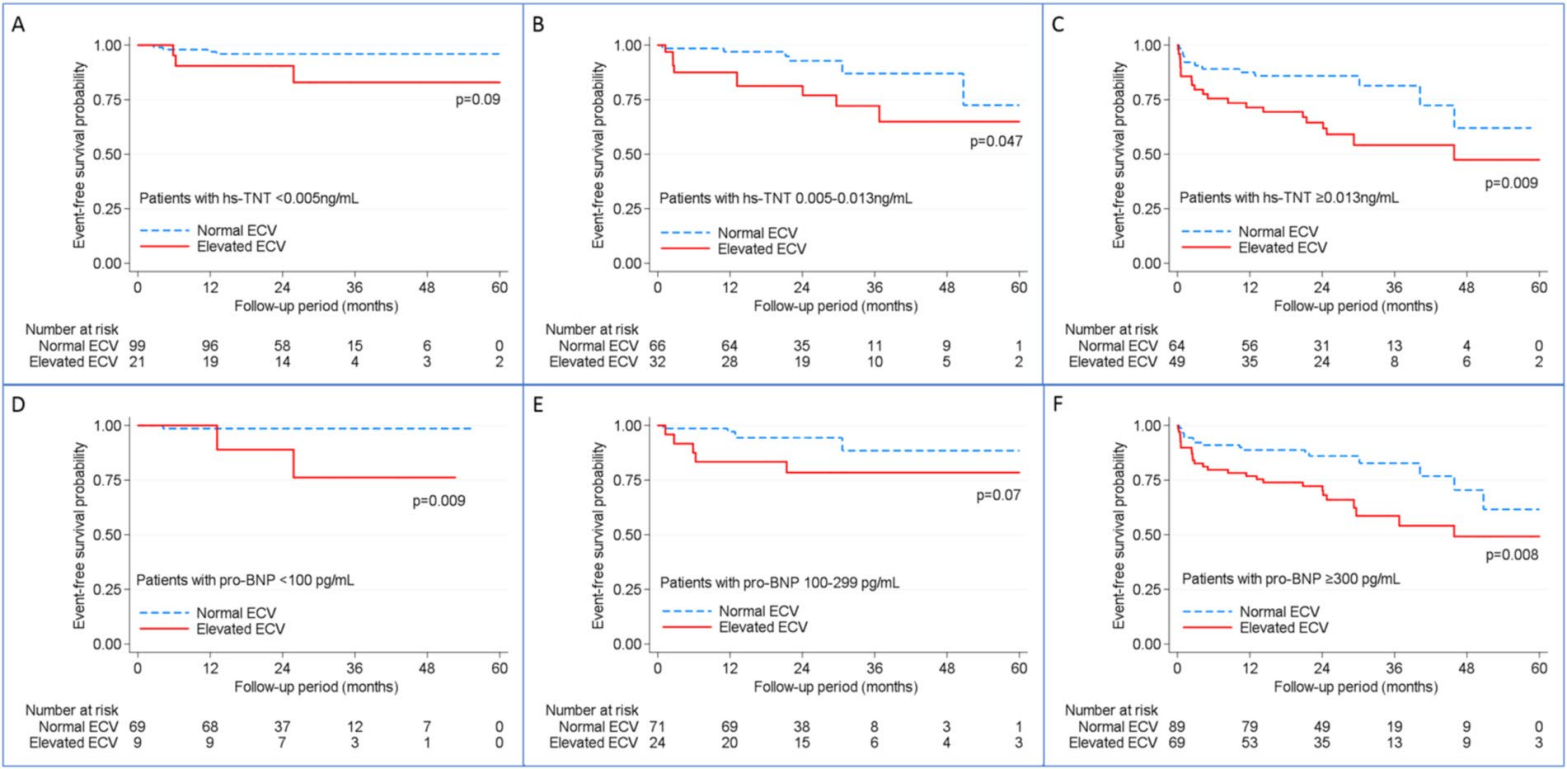
Kaplan-Meier survival curves are shown demonstrating event-free survival from a first composite heart failure event stratified by normal (dashed blue) vs. elevated extracellular volume (ECV) (solid red). The cohort was separated by categories of high-sensitivity troponin levels (hs-TNT) (top row, **A–C**) and by categories of N-terminal pro B-type natriuretic peptides (pro-BNP) (bottom row, **D–F**) based on recommended commercial cutoff values.

Over a median follow-up of 25.0 [interquartile range 21.8, 31.7] months, there were 55 first composite events, 27 deaths, and 32 heart failure hospitalizations. Given the proportion of patients who died, had heart failure hospitalization or had composite event between the low versus high ECV groups were 5.7% versus 13.7%, 4.4% versus 21.6%, and 9.6% versus 32.3%, respectively, our sample size of 331 had 99% power to detect the significant difference in composite event and heart failure hospitalization between low and high ECV groups. However, our sample had only 70% power in detecting the difference in mortality between ECV groups. Participants in categories with the greatest levels of hs-cTnT and NT-proBNP had reduced event-free survival from a first composite heart failure event, if they had elevated scar-free ECV (Table 7, Fig. 1, Supplemental Figs. 3 and 4). Similar trends were noted for low and intermediate levels of biomarkers even after minimal adjustments for covariates. The number of composite heart failure events within these biomarkers categories was small (Table 7). The event rates and hazard ratios for the primary outcome and event subtypes by biomarker categories can be found in Supplemental Table 4.

**Table 7:** Risk of the composite event, stratified by categories of NT-proBNP and high-sensitivity troponin T level.

	Normal ECV		Elevated ECV		Cox proportional hazards model (for elevated ECV vs. normal ECV)			
	Event/At risk, n	Incidence rate (per 100 person-years)	Event/At risk, n	Incidence rate (per 100 person-years)	Unadjusted		Adjusted**	
					HR (95% CI)	p-value	HR (95% CI)	p-value
**Composite event**								
NT-proBNP level (pg/mL)								
<100	1/69	0.63	2/9	7.96	12.61 (1.12, 141.93)	0.04	11.23 (0.87, 144.80)	0.06
100 to 299	5/71	3.19	5/24	8.40	3.01 (0.87, 10.48)	0.08	3.01 (0.81, 11.23)	0.10
≥300	16/89	8.01	26/69	18.47	2.27 (1.22, 4.23)	0.01	2.28 (1.21, 4.29)	0.01
High-sensitivity troponin level (ng/mL)								
<0.005	4/99	1.79	3/21	5.73	3.41 (0.76, 15.29)	0.11	4.22 (0.79, 22.47)	0.09
0.005 to 0.013	6/66	3.90	9/32	11.43	2.75 (0.97, 7.80)	0.06	2.65 (0.92, 7.67)	0.07
≥0.014	12/64	8.75	21/49	22.27	2.49 (1.22, 5.07)	0.01	2.42 (1.17, 5.03)	0.02

*Absolute risk difference is defined as the incidence rate difference (95% CI) between elevated ECV and normal ECV groups.

**Adjusted for age, gender and black ethnicity in multivariable models.

NT-proBNP, N-terminal pro B-type natriuretic peptide; ECV, extracellular volume; HR, hazards ratio; CI, confidence interval.

When imaging and blood marker variables were successively added to a heart failure model, only models containing imaging markers had a significant improvement in c-statistic for detecting a first composite event (Supplemental Tables 5–10). In fact, although the c-statisic improvement was not significant, when ECV was added to a full model including other imaging parameters and scar, its addition resulted in net reclassification indices of 0.670 (95% CI 0.274, 0.902) as a binary variable and of 0.536 (0.141, 0.868) as a continuous variable (Supplemental Table 10). The integrated discrimination index was not significant for ECV as a binary variable (IDI 0.039 [95% CI −0.000, 0.100] but was significant for ECV as a continuous variable (IDI 0.042 [95% CI 0.001, 0.100]). When stratified by presence of preserved LV systolic function (i.e., LV ejection fraction ≥50%, n = 240), imaging markers improved the model only for detecting mortality in participants with preserved LV systolic function (Supplemental Tables 11–14). However, only the presence of elevated ECV maintained a significant association with all heart failure outcomes in the full model with both imaging and blood biomarkers (Supplemental Table 14). No such relationship was observed between imaging and blood markers with heart failure outcomes in patients with LV ejection fraction <50% (Supplemental Tables 15–18).

## Discussion

We examined the relationship of ECV, hs-cTnT, and NT-proBNP measures with incident heart failure events. The principal findings of this study are as follows: a) hs-cTnT and NT-proBNP levels have modest associations with elevated ECV, more so with scar presence, that persist despite adjustments for relevant covariates in this population; and b) elevated ECV was associated with increased risk for first composite events for patients in the highest category levels for both blood biomarkers and for intermediate levels of hs-cTnT. For first composite events, a trend for significance was observed with elevated ECV for intermediate levels of NT-proBNP and the lowest category levels of both blood biomarkers.

Non-invasive quantification of ECV has been histologically validated in various small studies of patients with cardiac diseases^4^. Prior investigators have also examined the associations of CMR-derived imaging markers with hs-cTnT and NT pro-BNP levels. Chin *et al*. observed that patients with aortic stenosis had elevated hs-cTnT levels with mid-wall LGE present vs. those without LGE and also with increasing ECV values in fully adjusted models. In contrast, BNP levels were not associated with either CMR imaging marker in their cohort after adjustments^28^. In a study of patients with hypertrophic cardiomyopathy, Goh *et al*. also found that elevated hs-cTnT levels were similarly associated with mid-wall LGE presence vs. those without LGE and also with increasing interstitial volume after adjusting for covariates. Unlike their previous cohort with aortic stenosis, the same investigators found a similar relationship between these CMR-derived imaging markers and BNP in their cohort with hypertrophic cardiomyopathy, persisting after adjustments^29^. Our findings that these CMR imaging markers are associated with cardiac troponin and natriuretic peptide levels are consistent with both works by the same group of investigators but in a more general CMR referral base in a clinical setting.

To date, only a few large single center studies have established the association of ECV values with incident heart failure events^1–5^. Schelbert *et al*. have shown that ECV measures were strongly associated with log-transformed BNP levels in a subcohort of patients (*n* = 397) with heart failure with preserved ejection fraction or at risk for heart failure (univariable β linear regression coefficient = 0.338 [standard error (SE) 0.050], p < 0.001; multivariable β = 0.254 (SE 0.053], p < 0.001)^5^. Although Kammerlander *et al*. also observed an association between NT-proBNP and heart failure outcomes (HR 1.812 [95% CI 1.431, 2.294], p < 0.001) in their Austrian cohort (n = 473), this association did not persist in a multivariable model inclusive of ECV measures, which itself remained a significant predictor. Our univariable and multivariable models echo the findings of both observational studies, with elevated ECV persisting as a predictor of incident heart failure events. Additionally, when we examined improvement statistics, we found ECV to have significant improvement for detecting first composite heart failure events when added to a full heart failure prediction model plus LV EF and myocardial mass, hs-cTnT and NT pro-BNP, and myocardial scar. To our knowledge, few studies have also examined the relationship of ECV measures on CMR with troponin levels measured with high sensitivity assays.

Authors of the American College of Cardiology Foundation/American Heart Association Guideline for the Management of Heart Failure recognized the utility of various biomarkers for assessing patients with congestive heart failure^6,8^. For natriuretic peptides, the writing group recognized in their 2017 focused update that these markers may also have utility in screening populations for incident heart failure as well. Cardiac troponin levels using conventional assays were similarly commented by the writing group to be suggestive of cardiomyocyte injury or necrosis and should be interpreted within a given clinical context. However, authors of a recent meta-analysis of high sensitivity troponin measures in 154,052 individuals suggest other causes, such as atrial fibrillation, subclinical coronary ischemia, and even neurohormonal stressors may also play a role in their elevation^9^.

The same heart failure guideline writing group also identified several emerging blood biomarkers of interest associated with myocardial tissue activity such as soluble ST2 and galectin-3 for possible roles in heart failure risk stratification and management. Except for assessing potential etiologies of heart failure such as cardiac ischemia or infiltrative cardiomyopathies, the writing group did not consider noninvasive imaging markers beyond ventricular morphology and function for further heart failure management. More pointedly, the writing group endorsed the consideration of additional biomarkers for myocardial injury or fibrosis for additive risk stratification as a class IIB recommendation. Further, a need was identified for future multicenter studies that employ strategies combining multiple biomarkers for guiding heart failure therapies. Our study would be among the first to examine an imaging marker of myocardial fibrosis in the context of established blood markers of myocardial stress and injury in relation to the prognostic risk of heart failure outcomes. The work also provides a premise for framing ECV with such blood markers in future heart failure studies.

### Limitations

The study was not without limitations. As an observational cohort study, we included many demographic variables associated with heart failure outcomes at baseline, but unanticipated variables associated with heart failure outcomes may not have been captured at initial enrollment. Every reasonable effort was made to determine the vital status of participants and to contact participants regarding interval clinical events. However, outcomes of interest may still be missed despite our best efforts. The number of events were also small. Thus, we may not have been sufficiently powered to detect differences in outcomes associated with lower levels of blood biomarkers and ECV. Our findings should be construed as hypothesis-generating and would require further validation in a cohort with larger, sufficient event numbers. We also applied a model developed for a general population to a CMR referral base. Despite this issue, we still found that the ARIC heart failure risk model to have a reasonable discriminative ability for future heart failure outcomes in our population with c-statistics >0.70 for all studied outcomes, even before the addition of any additional risk markers to the risk models. Lastly, we used a CMR referral base at a tertiary care center, which may have a selection bias not only for more prevalent cardiac pathologies but also for less severe renal disease, because of the requirement for gadolinium contrast use. Thus, our study population may be less generalizable to other populations

## Conclusions

Elevated noninvasive measures of extracellular volume fraction on cardiac magnetic resonance imaging are associated with incident heart failure outcomes in patients with higher troponin T levels on high sensitivity assays and with higher N-terminal pro B-type natriuretic peptide levels. This imaging marker may have a role in providing additional risk stratification in patients at risk for heart failure and should be considered in future studies combining heart failure risk markers.

## Supplementary information

Supplementary Information
