# Supplementary material for: Relationship of extracellular volume assessed on cardiac magnetic resonance and serum cardiac troponins and natriuretic peptides with heart failure outcomes
Source: Sci Rep. 2019 Dec 27;9:20168. doi: 10.1038/s41598-019-56213-4 (PMC6934524; doi:10.1038/s41598-019-56213-4)
Supplement: Supplementary file 1 — Supplementary Information [file 41598_2019_56213_MOESM1_ESM.docx]

Relationship of extracellular volume assessed on cardiac magnetic resonance and serum cardiac troponins and natriuretic peptides with heart failure outcomes

Supplementary Information

## Authors:

Eric Y. Yang, MD, PhD^1^; Mohammad A. Khan, MD^1^; Edward A. Graviss, PhD, MPH^1^; Duc T. Nguyen, MD, PhD^1^; Arvind Bhimaraj, MD, MPH^1^; Vijay Nambi, MD, PhD^2,3,1^; Ron C. Hoogeveen, PhD^3^; Christie M. Ballantyne, MD^3,1^; William A. Zoghbi, MD^1^; Dipan J. Shah, MD^1*^

## Institutions:

^1^Houston Methodist Hospital – Houston, TX; ^2^Michael E. DeBakey Veterans Affairs Medical Center – Houston, TX; ^3^Department of Medicine, Baylor College of Medicine – Houston, TX

Supplemental Table 1. Indications for cardiac magnetic resonance studies.

| Indication | Frequency |
| --- | --- |
| CHF/Cardiomyopathy | 31.7% |
| Valve Assessment | 31.1% |
| Viability Assessment | 11.8% |
| Congenital Heart Disease | 9.4% |
| PV Ablation | 9.1% |
| Vascular Disease | 6.9% |
| Abnormal Echo | 5.7% |
| Arrhythmia | 4.8% |
| RV Dysplasia Evaluation | 3.9% |
| Ischemia Evaluation | 3.6% |
| Lung transplant | 3.6% |
| Cardiac Mass / Thrombus | 3.0% |
| HCM | 2.7% |
| Pericardial Evaluation | 2.7% |
| Abnormal ECG | 2.1% |
| Stroke Evaluation | 1.2% |
| Heart transplant | 0.9% |
| Anomalous Coronaries | 0.9% |
| Post-MI | 0.6% |
| Pre-Op Evaluation | 0.6% |
| Syncope | 0.3% |
| Aortic Dissection | 0.3% |

Supplemental Figure 1.

Patient Flow Diagram

**Supplemental Methods.**

Healthy Volunteers:

28 healthy volunteers (*n*=13, aged 35.5 [SD 5.2] years, 69% male on the 1.5 Tesla scanner; *n*=15, aged 39.5 [SD 7.8] years, 80% male on the 3.0 Tesla scanner) without any known cardiovascular diseases or risk factors were recruited to determine the distribution of extracellular volume fractions in a reference control group.

Supplemental Table 2. Correlation between NT proBNP, hs-cTnT with elevated ECV, as continuous and binary variables

|  | **rho*** | **p*** |
| --- | --- | --- |
| **Elevated ECV** |  |  |
| **Continuous biomarkers** |  |  |
| NT pro-BNP (pg/mL) and elevated ECV | 0.34 | <0.001 |
| High-sensitivity troponin (ng/mL) and elevated ECV | 0.26 | <0.001 |
| **Binary biomarkers** |  |  |
| Elevated NT pro-BNP (≥100pg/mL) and elevated ECV | 0.23 | <0.001 |
| Elevated high-sensitivity troponin (≥0.005ng/mL) and elevated ECV | 0.22 | <0.001 |
| **Any scar** |  |  |
| **Continuous biomarkers** |  |  |
| NT pro-BNP (pg/mL) and any scar | 0.47 | <0.001 |
| High-sensitivity troponin (ng/mL) and any scar | 0.48 | <0.001 |
| **Binary biomarkers** |  |  |
| Elevated NT pro-BNP (≥100pg/mL) and any scar | 0.37 | <0.001 |
| Elevated high-sensitivity troponin (≥0.005ng/mL) and any scar | 0.42 | <0.001 |
| ***Spearman correlation test** |  |  |

Supplemental Table 3. Association between ECV or any scar with elevated NT pro-BNP or high-sensitivity troponin, summary of multivariable linear regression

|  | **Continuous ECV (for each 5% increase)** | |
| --- | --- | --- |
|  | **Adjusted β coefficient (95% CI)** | **p-value** |
| **Continuous NT pro-BNP and high-sensitivity troponin** |  |  |
| NT pro-BNP (pg/mL) | 0.001 (-0.002, 0.004) | 0.38 |
| High-sensitivity troponin (ng/mL)*1000 | 0.0004 (-0.001, 0.002) | 0.40 |
| **Binary NT pro-BNP and high-sensitivity troponin** |  |  |
| Elevated NT pro-BNP (≥100pg/mL) | 0.24 (0.04, 0.43) | 0.02 |
| Elevated high-sensitivity troponin (≥0.005 ng/mL) | 0.23 (0.05, 0.42) | 0.02 |
| **Categorical NT pro-BNP and high-sensitivity troponin** |  |  |
| NT pro-BNP level (pg/mL) |  |  |
| <100 |  |  |
| 100 to 299 | 0.17 (-0.04, 0.38) | 0.11 |
| ≥300 | 0.32 (0.09, 0.55) | 0.01 |
| High sensitivity troponin T level (ng/mL) |  |  |
| <0.005 |  |  |
| 0.005 to 0.013 | 0.18 (-0.02, 0.38) | 0.09 |
| ≥ 0.014 | 0.22 (-0.03, 0.46) | 0.08 |

OR = odds ratio; ECV = extracellular volume; NT pro-BNP = N-terminal pro B-type natriuretic peptide

*Multiple logistic regression model includes NT pro-BNP, high-sensitivity troponin, age (years), gender, black (versus non-black), history of diabetes, treatment with diuretics, treatment with statin

Supplemental Figure 2.

Association between ECV or any scar with elevated NT pro-BNP or high-sensitivity troponin

ECV, extracellular volume; Elevated NT pro-BNP: ≥100pg/mL; Elevated high-sensitivity troponin: ≥0.005 ng/mL

For NT pro-BNP: Elevated, ≥100pg/mL; Normal, <100pg/mL

For high-sensitivity troponin T: Elevated, ≥0.005 ng/mL; Normal, <0.005 ng/mL

**Comparison testing (p-values) was performed using the Kruskal-Wallis test**

Supplemental Table 4. Risk of the composite event, stratified by categories of NT pro-BNP and high sensitivity troponin T level

|  | **Normal ECV** | | **Elevated ECV** | | **Cox proportional hazards model (for elevated ECV vs. normal ECV)** | | | |
| --- | --- | --- | --- | --- | --- | --- | --- | --- |
|  | **Event/At risk, n** | **Incidence rate** | **Event/At risk, n** | **Incidence rate** | **Unadjusted** | | **Adjusted**** | |
|  |  | **(per 100 person-years)** |  | **(per 100 person-years)** | **HR (95% CI)** | **p-value** | **HR (95% CI)** | **p-value** |
| **Composite event** |  |  |  |  |  |  |  |  |
| NT pro-BNP level (pg/mL) |  |  |  |  |  |  |  |  |
| <100 | 1/69 | 0.63 | 2/9 | 7.96 | 12.61 (1.12, 141.93) | 0.04 | 11.23 (0.87, 144.80) | 0.06 |
| 100 to 299 | 5/71 | 3.19 | 5/24 | 8.40 | 3.01 (0.87, 10.48) | 0.08 | 3.01 (0.81, 11.23) | 0.10 |
| ≥300 | 16/89 | 8.01 | 26/69 | 18.47 | 2.27 (1.22, 4.23) | 0.01 | 2.28 (1.21, 4.29) | 0.01 |
| High sensitivity troponin T level (ng/mL) |  |  |  |  |  |  |  |  |
| <0.005 | 4/99 | 1.79 | 3/21 | 5.73 | 3.41 (0.76, 15.29) | 0.11 | 4.22 (0.79, 22.47) | 0.09 |
| 0.005 to 0.013 | 6/66 | 3.90 | 9/32 | 11.43 | 2.75 (0.97, 7.80) | 0.06 | 2.65 (0.92, 7.67) | 0.07 |
| ≥ 0.014 | 12/64 | 8.75 | 21/49 | 22.27 | 2.49 (1.22, 5.07) | 0.01 | 2.42 (1.17, 5.03) | 0.02 |
| **Death** |  |  |  |  |  |  |  |  |
| NT pro-BNP level (pg/mL) |  |  |  |  |  |  |  |  |
| <100 | 0/69 | 0.00 | 1/9 | 3.83 | NA |  | NA |  |
| 100 to 299 | 2/71 | 1.25 | 3/24 | 4.43 | 4.60 (0.77, 27.51) | 0.10 | 4.83 (0.72, 32.56) | 0.11 |
| ≥300 | 11/89 | 5.26 | 10/69 | 5.79 | 1.10 (0.47, 2.59) | 0.83 | 1.07 (0.43, 2.42) | 0.97 |
| High sensitivity troponin T level (ng/mL) |  |  |  |  |  |  |  |  |
| <0.005 | 2/99 | 0.88 | 2/21 | 3.66 | 4.31 (0.60, 30.91) | 0.15 | 13.45 (1.43, 126.37) | 0.02 |
| 0.005 to 0.013 | 3/66 | 1.91 | 1/32 | 1.08 | 0.57 (0.06, 5.51) | 0.62 | 0.32 (0.03, 3.55) | 0.36 |
| ≥ 0.014 | 8/64 | 5.57 | 11/49 | 9.18 | 1.65 (0.66, 4.11) | 0.28 | 1.45 (0.56, 3.74) | 0.45 |
| **Heart failure hospitalization** |  |  |  |  |  |  |  |  |
| NT pro-BNP level (pg/mL) |  |  |  |  |  |  |  |  |
| <100 | 1/69 | 0.63 | 1/9 | 3.98 | 7.61 (0.48, 151.68) | 0.15 | 4.59 (0.20, 103.18) | 0.34 |
| 100 to 299 | 3/71 | 1.91 | 3/24 | 5.04 | 2.79 (0.56, 13.97) | 0.21 | 2.31 (0.41, 12.96) | 0.34 |
| ≥300 | 6/89 | 3.00 | 18/69 | 12.79 | 4.24 (1.68, 10.68) | 0.002 | 4.30 (1.69, 10.91) | 0.002 |
| High sensitivity troponin T level (ng/mL) |  |  |  |  |  |  |  |  |
| <0.005 | 2/99 | 0.90 | 1/21 | 1.91 | 2.33 (0.21, 25.73) | 0.49 | 1.67 (13.98, 20.03) | 0.68 |
| 0.005 to 0.013 | 3/66 | 1.95 | 9/32 | 11.43 | 5.56 (1.49, 20.73) | 0.01 | 5.64 (1.49, 21.37) | 0.01 |
| ≥ 0.014 | 5/64 | 3.65 | 12/49 | 12.73 | 3.44 (1.21, 9.77) | 0.02 | 3.30 (1.14, 9.57) | 0.03 |
| *Absolute risk difference is defined as the incidence rate difference (95% CI) between elevated ECV and normal ECV groups. | | | | | | | |  |
| **Adjusted for age, gender and Black race. | | |  |  |  |  |  |  |

Supplemental Table 5. Multivariable model comparisons for discriminating heart failure events by c-statistic

| **All patients** | | | | | | |
| --- | --- | --- | --- | --- | --- | --- |
|  | **First composite event (n=55/331)** | | **Death (n=27/331)** | | **Heart failure hospitalization (n=32/331)** | |
|  | **C-statistic (95% CI)** | **p-value*** | **C-statistic (95% CI)** | **p-value*** | **C-statistic (95% CI)** | **p-value*** |
| **[1] Original heart failure model only (no ECV, no biomarkers)** | 0.70 (0.63, 0.77) | (ref) | 0.79 (0.71, 0.87) | (ref) | 0.70 (0.62, 0.79) | (ref) |
| **[2] Original model + imaging markers (no biomarkers)** |  |  |  |  |  |  |
| 2.1. Original model + elevated ECV | 0.75 (0.69, 0.82) | 0.03 | 0.80 (0.72, 0.87) | 0.20 | 0.77 (0.68, 0.86) | 0.03 |
| 2.2. Original model + elevated ECV + scar presence | 0.75 (0.68, 0.82) | 0.047 | 0.80 (0.72, 0.87) | 0.38 | 0.78 (0.70, 0.87) | 0.02 |
| 2.3. Original model + elevated ECV + scar presence + LVEF | 0.76 (0.70, 0.83) | 0.02 | 0.80 (0.72, 0.87) | 0.44 | 0.81 (0.74, 0.88) | 0.001 |
| 2.4. Original model + elevated ECV + scar presence + LVEF + LV mass index | 0.77 (0.71, 0.84) | 0.01 | 0.78 (0.70, 0.86) | 0.89 | 0.81 (0.74, 0.89) | 0.001 |
| **[3] Original model + biomarkers (no imaging markers)** |  |  |  |  |  |  |
| 3.1. Original model + NT Pro-BNP (each 100 pg/mL increase) | 0.72 (0.64, 0.79) | 0.18 | 0.80 (0.72, 0.88) | 0.35 | 0.73 (0.65, 0.82) | 0.17 |
| 3.2. Original model + hs-TNT (ng/mL)*1000 | 0.71 (0.64, 0.78) | 0.30 | 0.79 (0.71, 0.87) | 0.81 | 0.71 (0.62, 0.80) | 0.56 |
| 3.3. Original model + NT Pro-BNP (each 100 pg/mL increase) + hs-TNT (ng/mL)*1000 | 0.72 (0.64, 0.79) | 0.18 | 0.80 (0.72, 0.88) | 0.31 | 0.73 (0.65, 0.82) | 0.23 |
| **[4] Original model + imaging markers + biomarkers**** | 0.77 (0.70, 0.84) | 0.02 | 0.80 (0.71, 0.88) | 0.46 | 0.83 (0.75, 0.90) | <0.001 |
| **LV-EF ≥50% cohort** | | | | | | |
|  | **First composite event (n=28/241)** | | **Death (n=16/241)** | | **Heart failure hospitalization (n=14/241)** | |
|  | **C-statistic (95% CI)** | **p-value*** | **C-statistic (95% CI)** | **p-value*** | **C-statistic (95% CI)** | **p-value*** |
| **[1] Original heart failure model only (no ECV, no biomarkers)** | 0.72 (0.62, 0.83) | (ref) | 0.76 (0.64, 0.88) | (ref) | 0.80 (0.69, 0.91) | (ref) |
| **[2] Original model + imaging markers (no biomarkers)** |  |  |  |  |  |  |
| 2.1. Original model + elevated ECV | 0.77 (0.66 0.87) | 0.26 | 0.83 (0.72, 0.94) | 0.045 | 0.83 (0.71, 0.95) | 0.31 |
| 2.2. Original model + elevated ECV + scar presence | 0.76 (0.66, 0.87) | 0.30 | 0.83 (0.72, 0.94) | 0.046 | 0.83 (0.71, 0.95) | 0.34 |
| 2.3. Original model + elevated ECV + scar presence + LVEF | 0.76 (0.65, 0.87) | 0.33 | 0.83 (0.72, 0.94) | 0.046 | 0.83 (0.71, 0.95) | 0.34 |
| 2.4. Original model + elevated ECV + scar presence + LVEF + LV mass index | 0.77 (0.71, 0.84) | 0.20 | 0.83 (0.72, 0.94) | 0.047 | 0.85 (0.72, 0.98) | 0.14 |
| **[3] Original model + biomarkers (no imaging markers)** |  |  |  |  |  |  |
| 3.1. Original model + NT Pro-BNP (each 100 pg/mL increase) | 0.74 (0.64, 0.84) | 0.35 | 0.80 (0.68, 0.91) | 0.16 | 0.82 (0.72, 0.93) | 0.06 |
| 3.2. Original model + hs-TNT (ng/mL)*1000 | 0.72 (0.62, 0.83) | 0.78 | 0.76 (0.64, 0.87) | 0.49 | 0.80 (0.69, 0.91) | 0.42 |
| 3.3. Original model + NT Pro-BNP (each 100 pg/mL increase) + hs-TNT (ng/mL)*1000 | 0.74 (0.65, 0.84) | 0.34 | 0.80 (0.68, 0.91) | 0.16 | 0.82 (0.71, 0.93) | 0.054 |
| **[4] Original model + imaging markers + biomarkers**** | 0.78 (0.67, 0.89) | 0.31 | 0.84 (0.72, 0.96) | 0.06 | 0.85 (0.72, 0.98) | 0.20 |
| **LV-EF <50% cohort** | | | | | | |
|  | **First composite event (n=27/90)** | | **Death (n=11/90)** | | **Heart failure hospitalization (n=18/90)** | |
|  | **C-statistic (95% CI)** | **p-value*** | **C-statistic (95% CI)** | **p-value*** | **C-statistic (95% CI)** | **p-value*** |
| **[1] Original heart failure model only (no ECV, no biomarkers)** | 0.73 (0.63, 0.83) | (ref) | 0.84 (0.76, 0.93) | (ref) | 0.73 (0.61, 0.85) | (ref) |
| **[2] Original model + imaging markers (no biomarkers)** |  |  |  |  |  |  |
| 2.1. Original model + elevated ECV | 0.75 (0.65, 0.85) | 0.39 | 0.84 (0.74, 0.94) | 0.68 | 0.78 (0.66, 0.89) | 0.26 |
| 2.2. Original model + elevated ECV + scar presence | 0.75 (0.65, 0.85) | 0.42 | 0.84 (0.74, 0.94) | 0.68 | 0.78 (0.67, 0.89) | 0.26 |
| 2.3. Original model + elevated ECV + scar presence + LVEF | 0.75 (0.65, 0.85) | 0.30 | 0.85 (0.75, 0.95) | 0.82 | 0.78 (0.67, 0.89) | 0.20 |
| 2.4. Original model + elevated ECV + scar presence + LVEF + LV mass index | 0.76 (0.66, 0.86) | 0.26 | 0.85 (0.74, 0.96) | 0.97 | 0.77 (0.66, 0.89) | 0.20 |
| **[3] Original model + biomarkers (no imaging markers)** |  |  |  |  |  |  |
| 3.1. Original model + NT Pro-BNP (each 100 pg/mL increase) | 0.74 (0.64, 0.84) | 0.37 | 0.84 (0.76, 0.93) | 0.48 | 0.74 (0.61, 0.86) | 0.74 |
| 3.2. Original model + hs-TNT (ng/mL)*1000 | 0.75 (0.64, 0.85) | 0.39 | 0.84 (0.75, 0.93) | 1.00 | 0.73 (0.60, 0.86) | 0.90 |
| 3.3. Original model + NT Pro-BNP (each 100 pg/mL increase) + hs-TNT (ng/mL)*1000 | 0.75 (0.64, 0.86) | 0.34 | 0.84 (0.75, 0.93) | 1.00 | 0.75 (0.63, 0.88) | 0.47 |
| **[4] Original model + imaging markers + biomarkers**** | 0.78 (0.67, 0.88) | 0.14 | 0.84 (0.71, 0.97) | 0.76 | 0.79 (0.67, 0.91) | 0.14 |

hs-TNT, high-sensitivity troponin; *C-statistic comparison: comparing the prognostic significance (C-statistic) of the modified models with that of the original model using the lincom function;
Original heart failure model: age, gender, black race, smoker, heart rate, BMI, prior diabetes, prior hypertension, prior myocardial infarction; **[4] Original model + imaging markers + biomarkers: Original heart failure model + ECV + scar presence + LVEF + LV mass index + hs-TnT + NT pro-BNP

Supplemental Table 6. Multivariable Cox proportional hazard risk models for the entire cohort using original heart failure risk variables

|  | **First composite event** | | **Mortality** | | **Heart Failure Hospitalization** | |
| --- | --- | --- | --- | --- | --- | --- |
|  | **Adjusted HR (95% CI)** | **p-value** | **Adjusted HR (95% CI)** | **p-value** | **Adjusted HR (95% CI)** | **p-value** |
| Age | 1.03 (1.01, 1.06) | 0.01 | 1.03 (0.99, 1.07) | 0.12 | 1.03 (1.00, 1.06) | 0.04 |
| Male gender | 1.25 (0.70, 2.22) | 0.45 | 1.52 (0.65, 3.58) | 0.34 | 0.83 (0.40, 1.71) | 0.62 |
| Black | 1.35 (0.67, 2.73) | 0.40 | 1.25 (0.46, 3.42) | 0.66 | 1.85 (0.78, 4.43) | 0.17 |
| BMI | 0.97 (0.93, 1.02) | 0.25 | 0.93 (0.87, 1.00) | 0.07 | 0.98 (0.93, 1.04) | 0.59 |
| Smoking | 1.09 (0.62, 1.92) | 0.76 | 1.13 (0.51, 2.51) | 0.77 | 0.87 (0.41, 1.87) | 0.73 |
| History of hypertension | 1.62 (0.75, 3.52) | 0.22 | 5.34 (1.12, 25.50) | 0.04 | 1.04 (0.41, 2.62) | 0.94 |
| History of diabetes | 1.66 (0.91, 3.01) | 0.10 | 1.59 (0.70, 3.59) | 0.27 | 1.58 (0.70, 3.59) | 0.28 |
| Heart rate | 1.02 (1.00, 1.04) | 0.02 | 1.04 (1.01, 1.07) | 0.01 | 1.01 (0.98, 1.04) | 0.47 |
| History of myocardial infarction | 1.00 (0.46, 2.15) | 1.00 | 1.42 (0.52, 3.87) | 0.49 | 0.83 (0.27, 2.50) | 0.74 |
| **C-statistic (95% CI)** | **0.70 (0.63, 0.77)** |  | **0.79 (0.71, 0.87)** |  | **0.70 (0.62, 0.79)** |  |

Supplemental Table 7. Multivariable Cox proportional hazard risk models for the entire cohort using original heart failure risk variables plus imaging markers

|  | **First composite event** | | **Mortality** | | **Heart Failure Hospitalization** | |
| --- | --- | --- | --- | --- | --- | --- |
|  | **Adjusted HR (95% CI)** | **p-value** | **Adjusted HR (95% CI)** | **p-value** | **Adjusted HR (95% CI)** | **p-value** |
| Age | 1.03 (1.00, 1.05) | 0.03 | 1.04 (1.00, 1.08) | 0.08 | 1.03 (0.99, 1.06) | 0.13 |
| Male gender | 0.89 (0.48, 1.65) | 0.71 | 1.55 (0.60, 4.00) | 0.36 | 0.45 (0.21, 1.01) | 0.052 |
| Black | 1.02 (0.49, 2.09) | 0.96 | 1.34 (0.48, 3.75) | 0.58 | 1.09 (0.44, 2.70) | 0.85 |
| BMI | 0.96 (0.92, 1.01) | 0.16 | 0.94 (0.87, 1.01) | 0.09 | 0.97 (0.91, 1.03) | 0.28 |
| Smoking | 1.00 (0.55, 1.83) | 1.00 | 1.20 (0.50, 2.85) | 0.69 | 0.72 (0.32, 1.62) | 0.43 |
| History of hypertension | 1.62 (0.71, 3.69) | 0.25 | 4.71 (0.96, 23.00) | 0.06 | 1.10 (0.39, 3.08) | 0.86 |
| History of diabetes | 1.28 (0.69, 2.40) | 0.43 | 1.40 (0.58, 3.38) | 0.46 | 1.43 (0.61, 3.36) | 0.41 |
| Heart rate | 1.02 (1.00, 1.04) | 0.09 | 1.04 (1.01, 1.07) | 0.01 | 1.00 (0.97, 1.02) | 0.74 |
| History of myocardial infarction | 0.58 (0.24, 1.41) | 0.23 | 1.39 (0.44, 4.36) | 0.58 | 0.35 (0.11, 1.18) | 0.09 |
| Elevated ECV | 2.64 (1.45, 4.81) | 0.002 | 1.28 (0.53, 3.08) | 0.58 | 3.79 (1.69, 8.53) | 0.001 |
| Any scar presence | 1.26 (0.64, 2.45) | 0.50 | 0.78 (0.31, 1.97) | 0.60 | 1.82 (0.76, 4.37) | 0.18 |
| LVEF | 0.99 (0.98, 1.01) | 0.54 | 1.01 (0.98, 1.03) | 0.66 | 0.98 (0.96, 1.00) | 0.09 |
| LV mass index | 1.01 (1.00, 1.02) | 0.04 | 1.00 (0.99, 1.02) | 0.67 | 1.01 (1.00, 1.03) | 0.14 |
| **C-statistic (95% CI)** | **0.77 (0.71, 0.84)** |  | **0.78 (0.70, 0.86)** |  | **0.81 (0.74, 0.89)** |  |

Supplemental Figure 3.


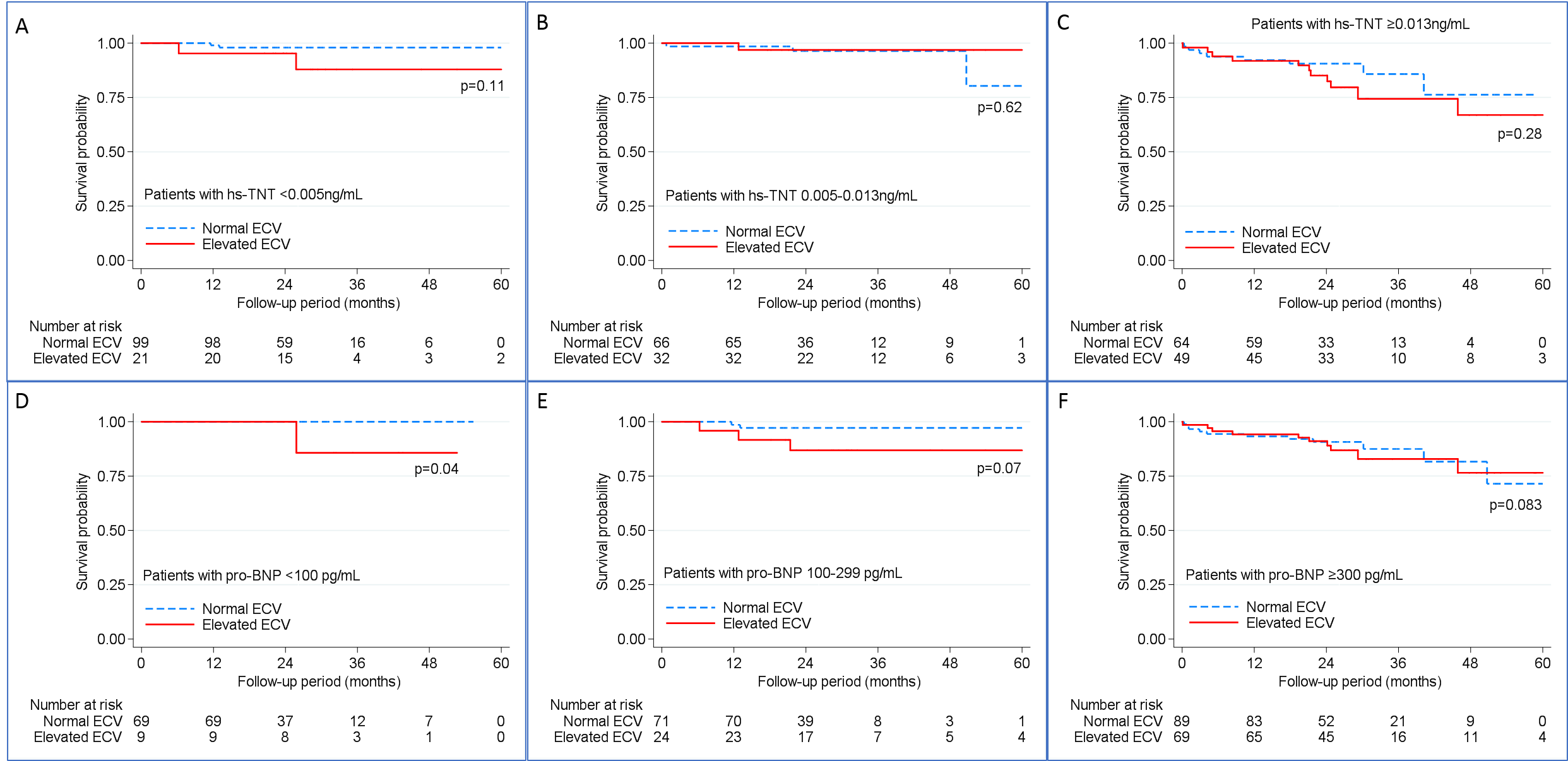


Kaplan-Meier survival curves are shown demonstrating event-free survival from all-cause death stratified by normal (blue) vs. elevated ECV (red). The cohort was separated by categories of high sensitivity troponin T levels (top row, A-C) and by categories of N-terminal pro B-type natriuretic peptides (bottom row, D-F) based on recommended commercial cutoff values.

Supplemental Figure 4.


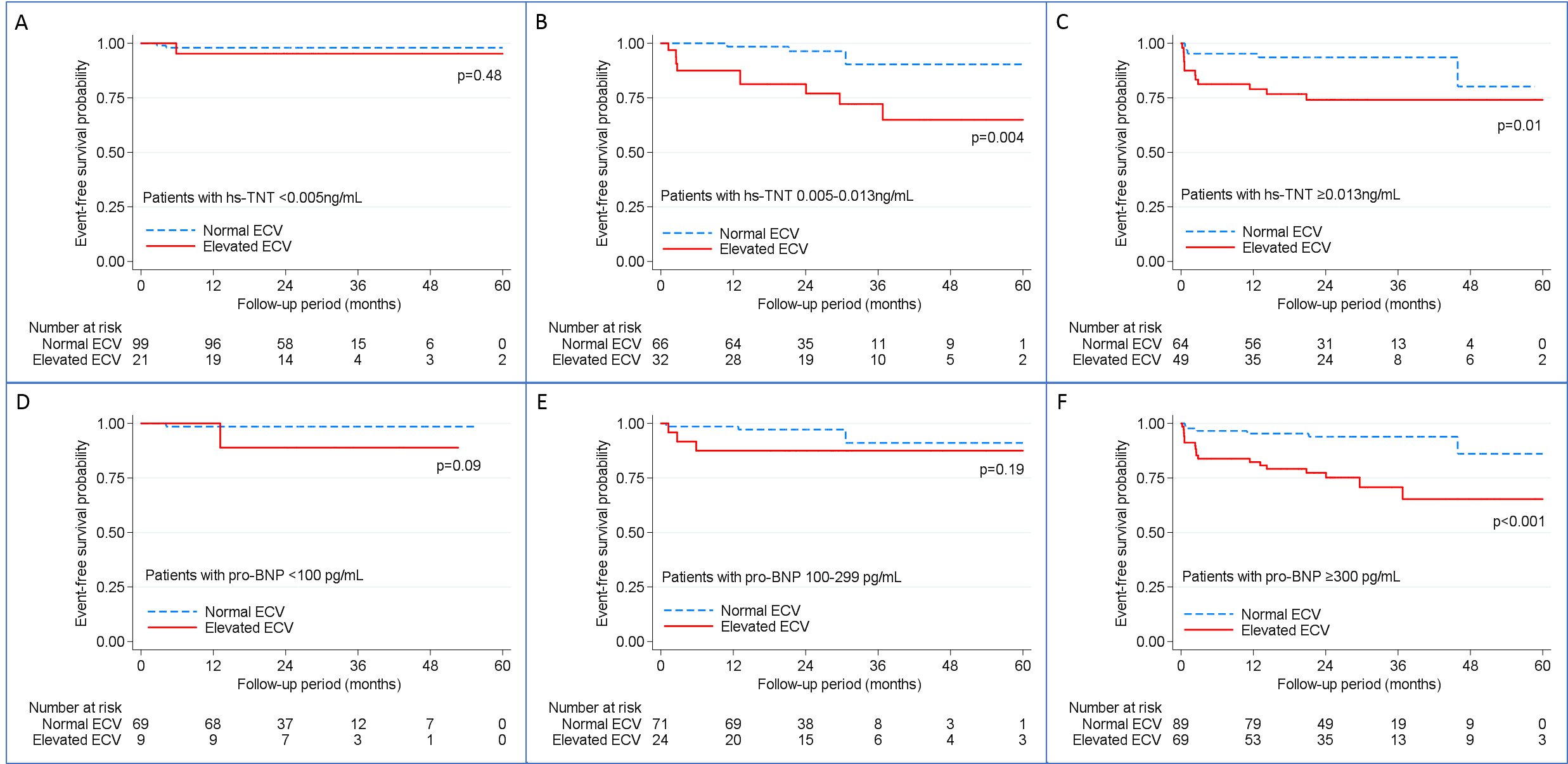


Kaplan-Meier survival curves are shown demonstrating event-free survival from heart failure hospitalization stratified by normal (blue) vs. elevated ECV (red). The cohort was separated by categories of high sensitivity troponin T levels (top row, A-C) and by categories of N-terminal pro B-type natriuretic peptides (bottom row, D-F) based on recommended commercial cutoff values.

Supplemental Table 8. Multivariable Cox proportional hazard risk models for the entire cohort using original heart failure risk variables plus blood markers

|  | **First composite event** | | **Mortality** | | **Heart Failure Hospitalization** | |
| --- | --- | --- | --- | --- | --- | --- |
|  | **Adjusted HR (95% CI)** | **p-value** | **Adjusted HR (95% CI)** | **p-value** | **Adjusted HR (95% CI)** | **p-value** |
| Age | 1.03 (1.00, 1.05) | 0.02 | 1.02 (0.99, 1.06) | 0.20 | 1.03 (1.00, 1.06) | 0.08 |
| Male gender | 1.23 (0.68, 2.22) | 0.49 | 1.58 (0.65, 3.81) | 0.31 | 0.82 (0.39, 1.74) | 0.61 |
| Black | 0.94 (0.41, 2.15) | 0.88 | 0.85 (0.25, 2.91) | 0.80 | 1.26 (0.46, 3.50) | 0.65 |
| BMI | 0.99 (0.94, 1.03) | 0.53 | 0.94 (0.87, 1.01) | 0.11 | 1.00 (0.94, 1.05) | 0.95 |
| Smoking | 1.19 (0.67, 2.10) | 0.56 | 1.21 (0.54, 2.74) | 0.64 | 0.96 (0.44, 2.07) | 0.91 |
| History of hypertension | 1.68 (0.76, 3.69) | 0.20 | 5.37 (1.11, 25.93) | 0.04 | 1.07 (0.42, 2.74) | 0.89 |
| History of diabetes | 1.53 (0.83, 2.82) | 0.17 | 1.54 (0.67, 3.53) | 0.31 | 1.49 (0.65, 3.45) | 0.35 |
| Heart rate | 1.01 (0.99, 1.04) | 0.15 | 1.03 (1.00, 1.06) | 0.045 | 1.00 (0.97, 1.03) | 0.90 |
| History of myocardial infarction | 0.64 (0.27, 1.48) | 0.29 | 1.02 (0.35, 3.01) | 0.97 | 0.48 (0.14, 1.65) | 0.24 |
| NT Pro-BNP (each 100 pg/mL increase) | 1.01 (1.00, 1.01) | 0.003 | 1.01 (1.00, 1.01) | 0.08 | 1.01 (1.00, 1.02) | 0.01 |
| hs-TNT (ng/mL)*1000 | 1.00 (1.00, 1.00) | 0.03 | 1.00 (1.00, 1.01) | 0.56 | 1.00 (1.00, 1.01) | 0.09 |
| **C-statistic (95% CI)** | **0.72 (0.64, 0.79)** |  | **0.80 (0.72, 0.88)** |  | **0.73 (0.65, 0.82)** |  |

Supplemental Table 9. Multivariable Cox proportional hazard risk models for the entire cohort using original heart failure risk variables plus imaging and blood markers

|  | **First composite event** | | **Mortality** | | **Heart Failure Hospitalization** | |
| --- | --- | --- | --- | --- | --- | --- |
|  | **Adjusted HR (95% CI)** | **p-value** | **Adjusted HR (95% CI)** | **p-value** | **Adjusted HR (95% CI)** | **p-value** |
| Age | 1.02 (1.00, 1.05) | 0.09 | 1.03 (0.99, 1.07) | 0.15 | 1.02 (0.98, 1.05) | 0.31 |
| Male gender | 0.97 (0.51, 1.84) | 0.93 | 1.91 (0.72, 5.08) | 0.20 | 0.49 (0.22, 1.11) | 0.09 |
| Black | 0.75 (0.33, 1.70) | 0.49 | 0.89 (0.25, 3.17) | 0.86 | 0.81 (0.30, 2.23) | 0.69 |
| BMI | 0.98 (0.93, 1.03) | 0.43 | 0.95 (0.88, 1.03) | 0.23 | 0.98 (0.92, 1.05) | 0.58 |
| Smoking | 1.11 (0.60, 2.03) | 0.75 | 1.34 (0.55, 3.24) | 0.52 | 0.78 (0.35, 1.78) | 0.56 |
| History of hypertension | 1.66 (0.72, 3.79) | 0.23 | 4.71 (0.95, 23.38) | 0.06 | 1.11 (0.39, 3.13) | 0.85 |
| History of diabetes | 1.19 (0.62, 2.27) | 0.60 | 1.33 (0.54, 3.28) | 0.54 | 1.39 (0.58, 3.34) | 0.47 |
| Heart rate | 1.01 (0.99, 1.03) | 0.38 | 1.03 (1.00, 1.06) | 0.054 | 0.99 (0.96, 1.02) | 0.35 |
| History of myocardial infarction | 0.37 (0.14, 0.95) | 0.04 | 0.88 (0.26, 2.96) | 0.83 | 0.21 (0.06, 0.78) | 0.02 |
| Elevated ECV | 2.77 (1.53, 5.02) | 0.001 | 1.36 (0.57, 3.24) | 0.49 | 3.96 (1.77, 8.87) | 0.001 |
| Any scar presence | 1.42 (0.72, 2.81) | 0.31 | 1.00 (0.37, 2.68) | 1.00 | 2.08 (0.86, 5.03) | 0.11 |
| LVEF | 1.00 (0.98, 1.02) | 0.96 | 1.02 (0.99, 1.04) | 0.26 | 0.99 (0.96, 1.01) | 0.22 |
| LV mass index | 1.01 (1.00, 1.02) | 0.10 | 1.00 (0.98, 1.02) | 0.93 | 1.01 (0.99, 1.03) | 0.20 |
| NT Pro-BNP (each 100 pg/mL increase) | 1.01 (1.00, 1.02) | 0.002 | 1.01 (1.00, 1.02) | 0.03 | 1.01 (1.00, 1.02) | 0.01 |
| hs-TNT (ng/mL)*1000 | 1.00 (1.00, 1.00) | 0.09 | 1.00 (1.00, 1.01) | 0.30 | 1.00 (1.00, 1.01) | 0.49 |
| **C-statistic (95% CI)** | **0.77 (0.70, 0.84)** |  | **0.80 (0.71, 0.88)** |  | **0.83 (0.75, 0.90)** |  |

Supplemental Table 10. Cox proportional hazard models, associated c-statistic values, and reclassification indices with the addition of scar, binary ECV, or continuous ECV to a base model.

|  | **Model 1. First composite event, without scar nor ECV** | | **Model 2. First composite event, with any scar** | | **Model 3a. First composite event, with any scar + binary ECV** | | **Model 3b. First composite event, with any scar + continuous ECV** | | |
| --- | --- | --- | --- | --- | --- | --- | --- | --- | --- |
|  | **Adjusted HR (95% CI)** | **p-value** | **Adjusted HR (95% CI)** | **p-value** | **Adjusted HR (95% CI)** | **p-value** | **Adjusted HR (95% CI)** | **p-value** | |
| Age | 1.03 (1.00, 1.05) | 0.02 | 1.03 (1.00, 1.05) | 0.04 | 1.02 (1.00, 1.05) | 0.09 | 1.02 (1.00, 1.05) | 0.10 | |
| Male gender | 0.95 (0.51, 1.77) | 0.86 | 0.91 (0.48, 1.71) | 0.77 | 0.97 (0.51, 1.84) | 0.93 | 1.03 (0.55, 1.93) | 0.93 | |
| Black | 0.98 (0.94, 1.03) | 0.47 | 0.98 (0.94, 1.03) | 0.46 | 0.98 (0.93, 1.03) | 0.43 | 0.98 (0.94, 1.03) | 0.52 | |
| BMI | 0.92 (0.40, 2.10) | 0.84 | 0.88 (0.39, 2.02) | 0.77 | 0.75 (0.33, 1.70) | 0.49 | 0.70 (0.31, 1.61) | 0.40 | |
| Smoking | 1.04 (0.58, 1.87) | 0.90 | 0.97 (0.53, 1.75) | 0.91 | 1.11 (0.60, 2.03) | 0.75 | 1.09 (0.60, 1.98) | 0.79 | |
| History of hypertension | 1.23 (0.83, 1.83) | 0.30 | 1.24 (0.84, 1.85) | 0.28 | 1.29 (0.85, 1.95) | 0.23 | 1.34 (0.89, 2.04) | 0.17 | |
| History of diabetes | 1.45 (0.77, 2.70) | 0.25 | 1.39 (0.74, 2.61) | 0.30 | 1.19 (0.62, 2.27) | 0.60 | 1.23 (0.65, 2.34) | 0.52 | |
| Heart rate | 1.01 (0.99, 1.03) | 0.21 | 1.01 (0.99, 1.03) | 0.28 | 1.01 (0.99, 1.03) | 0.38 | 1.01 (0.99, 1.03) | 0.50 | |
| History of myocardial infarction | 0.55 (0.22, 1.34) | 0.19 | 0.48 (0.19, 1.18) | 0.11 | 0.37 (0.14, 0.95) | 0.04 | 0.36 (0.14, 0.92) | 0.03 | |
| LVEF | 0.99 (0.97, 1.01) | 0.33 | 0.99 (0.98, 1.01) | 0.49 | 1.00 (0.98, 1.02) | 0.96 | 1.00 (0.98, 1.02) | 0.94 | |
| LV mass index | 1.01 (1.00, 1.02) | 0.15 | 1.01 (0.99, 1.02) | 0.28 | 1.01 (1.00, 1.02) | 0.10 | 1.01 (1.00, 1.02) | 0.14 | |
| NT Pro-BNP (each 100 pg/mL increase) | 1.01 (1.00, 1.01) | 0.01 | 1.01 (1.00, 1.01) | 0.004 | 1.01 (1.00, 1.02) | 0.002 | 1.01 (1.00, 1.02) | 0.002 | |
| hs-TNT (ng/mL)*1000 | 1.00 (1.00, 1.00) | 0.08 | 1.00 (1.00, 1.00) | 0.10 | 1.00 (1.00, 1.00) | 0.09 | 1.00 (1.00, 1.00) | 0.10 | |
| Any scar presence | -- | -- | 1.59 (0.80, 3.14) | 0.18 | 1.42 (0.72, 2.81) | 0.31 | 1.38 (0.70, 2.74) | | 0.35 |
| Elevated ECV | -- | -- | -- | -- | 2.77 (1.53, 5.02) | 0.001 | -- | | -- |
| ECV (per 5% increase) | -- | -- | -- | -- | -- | -- | 1.88 (1.30, 2.73) | | 0.001 |
| C-statistic (95 %CI) | 0.74 (0.67, 0.81) | | 0.74 (0.67, 0.81) | | 0.77 (0.70, 0.84) | | 0.77 (0.70, 0.84) | | |
| C-statistic improvement (95% CI); p-value* | -- | | -0.001 (-0.021, 0.018); p=0.89 | | 0.036 (-0.001, 0.073); 0.059 | | 0.033 (-0.001, 0.067); p=0.059 | | |
| NRI (95% CI)* | -- | | 0.427 (-0.342, 0.673) | | 0.670 ( 0.274, 0.902) | | 0.536 ( 0.141, 0.868) | | |
| IDI (95% CI)* | -- | | 0.002 (-0.007, 0.039) | | 0.039 (-0.000, 0.100) | | 0.042 ( 0.001, 0.100) | | |

CI = confidence interval; NRI = Net Reclassification Improvement; IDI = Integrated Discrimination Improvement

*Pairwise comparisons (model 2 vs. model 1, model 3 vs. model 2 and model 4 vs. model 2); --, not applicable

Supplemental Table 11. Multivariable Cox proportional hazard risk models for the participants with LV ejection fraction ≥50% using original heart failure risk variables

|  | **First composite event** | | **Mortality** | | **Heart Failure Hospitalization** | |
| --- | --- | --- | --- | --- | --- | --- |
|  | **Adjusted HR (95% CI)** | **p-value** | **Adjusted HR (95% CI)** | **p-value** | **Adjusted HR (95% CI)** | **p-value** |
| Age | 1.02 (0.98, 1.06) | 0.28 | 1.02 (0.98, 1.08) | 0.34 | 1.02 (0.97, 1.08) | 0.40 |
| Male gender | 1.23 (0.54, 2.81) | 0.62 | 2.41 (0.75, 7.74) | 0.14 | 0.56 (0.17, 1.84) | 0.34 |
| Black | 3.44 (1.17, 10.12) | 0.03 | 2.54 (0.55, 11.65) | 0.23 | 5.36 (1.28, 22.35) | 0.02 |
| BMI | 0.97 (0.91, 1.03) | 0.36 | 0.93 (0.84, 1.02) | 0.13 | 0.97 (0.89, 1.06) | 0.48 |
| Smoking | 1.13 (0.51, 2.50) | 0.77 | 1.47 (0.53, 4.06) | 0.46 | 0.65 (0.17, 2.48) | 0.53 |
| History of hypertension | 2.60 (0.86, 7.89) | 0.09 | 3.77 (0.72, 19.77) | 0.12 | 2.05 (0.45, 9.27) | 0.35 |
| History of diabetes | 1.43 (0.58, 3.56) | 0.44 | 1.55 (0.47, 5.18) | 0.47 | 1.26 (0.30, 5.27) | 0.75 |
| Heart rate | 0.99 (0.96, 1.02) | 0.51 | 1.02 (0.98, 1.06) | 0.25 | 0.96 (0.92, 0.99) | 0.03 |
| History of myocardial infarction | 1.14 (0.33, 3.90) | 0.84 | 2.51 (0.68, 9.30) | 0.17 | 0.63 (0.08, 5.11) | 0.66 |
| **C-statistic (95% CI)** | **0.72 (0.62, 0.83)** |  | **0.76 (0.64, 0.88)** |  | **0.80 (0.69, 0.91)** |  |

Supplemental Table 12. Multivariable Cox proportional hazard risk models for the participants with LV ejection fraction ≥50% using original heart failure risk variables plus imaging markers

|  | **First composite event** | | **Mortality** | | **Heart Failure Hospitalization** | |
| --- | --- | --- | --- | --- | --- | --- |
|  | **Adjusted HR (95% CI)** | **p-value** | **Adjusted HR (95% CI)** | **p-value** | **Adjusted HR (95% CI)** | **p-value** |
| Age | 1.01 (0.97, 1.05) | 0.56 | 1.01 (0.96, 1.07) | 0.68 | 1.02 (0.96, 1.08) | 0.51 |
| Male gender | 1.10 (0.48, 2.52) | 0.83 | 2.73 (0.80, 9.34) | 0.11 | 0.47 (0.14, 1.61) | 0.23 |
| Black | 2.32 (0.72, 7.48) | 0.16 | 1.83 (0.35, 9.48) | 0.47 | 3.59 (0.70, 18.33) | 0.12 |
| BMI | 0.98 (0.92, 1.04) | 0.51 | 0.92 (0.83, 1.01) | 0.10 | 1.01 (0.92, 1.10) | 0.90 |
| Smoking | 1.06 (0.46, 2.46) | 0.89 | 1.68 (0.56, 5.03) | 0.35 | 0.56 (0.13, 2.39) | 0.44 |
| History of hypertension | 2.00 (0.61, 6.56) | 0.25 | 3.55 (0.60, 20.83) | 0.16 | 1.30 (0.24, 7.00) | 0.76 |
| History of diabetes | 0.84 (0.31, 2.32) | 0.74 | 1.01 (0.26, 3.96) | 0.99 | 0.74 (0.16, 3.48) | 0.70 |
| Heart rate | 0.99 (0.96, 1.02) | 0.68 | 1.03 (0.98, 1.07) | 0.24 | 0.95 (0.91, 1.00) | 0.07 |
| History of myocardial infarction | 1.15 (0.33, 4.08) | 0.83 | 2.53 (0.67, 9.47) | 0.17 | 0.69 (0.08, 6.19) | 0.74 |
| Elevated ECV | 4.33 (1.83, 10.20) | 0.001 | 3.90 (1.19, 12.75) | 0.02 | 5.42 (1.52, 19.39) | 0.01 |
| Any scar presence | 1.19 (0.52, 2.74) | 0.68 | 0.91 (0.30, 2.77) | 0.87 | 1.57 (0.46, 5.40) | 0.47 |
| LVEF | 1.01 (0.96, 1.05) | 0.73 | 1.01 (0.95, 1.07) | 0.86 | 1.00 (0.94, 1.08) | 0.89 |
| LV mass index | 1.02 (1.00, 1.03) | 0.06 | 1.00 (0.97, 1.03) | 0.96 | 1.02 (1.00, 1.04) | 0.03 |
| **C-statistic (95% CI)** | **0.77 (0.71, 0.84)** |  | **0.83 (0.72, 0.94)** |  | **0.85 (0.72, 0.98)** |  |

Supplemental Table 13. Multivariable Cox proportional hazard risk models for the participants with LV ejection fraction ≥50% using original heart failure risk variables plus blood markers

|  | **First composite event** | | **Mortality** | | **Heart Failure Hospitalization** | |
| --- | --- | --- | --- | --- | --- | --- |
|  | **Adjusted HR (95% CI)** | **p-value** | **Adjusted HR (95% CI)** | **p-value** | **Adjusted HR (95% CI)** | **p-value** |
| Age | 1.02 (0.98, 1.06) | 0.29 | 1.02 (0.97, 1.07) | 0.40 | 1.02 (0.97, 1.08) | 0.41 |
| Male gender | 1.25 (0.53, 2.94) | 0.61 | 2.46 (0.72, 8.36) | 0.15 | 0.58 (0.16, 2.03) | 0.39 |
| Black | 2.58 (0.77, 8.58) | 0.12 | 1.53 (0.23, 10.18) | 0.66 | 4.80 (1.07, 21.53) | 0.04 |
| BMI | 0.99 (0.93, 1.06) | 0.77 | 0.96 (0.87, 1.06) | 0.41 | 0.98 (0.90, 1.07) | 0.60 |
| Smoking | 1.29 (0.57, 2.92) | 0.54 | 1.74 (0.60, 5.02) | 0.31 | 0.71 (0.18, 2.76) | 0.62 |
| History of hypertension | 2.60 (0.86, 7.87) | 0.09 | 3.80 (0.71, 20.32) | 0.12 | 2.05 (0.45, 9.31) | 0.35 |
| History of diabetes | 1.14 (0.43, 3.06) | 0.79 | 1.14 (0.30, 4.35) | 0.85 | 1.11 (0.24, 5.08) | 0.89 |
| Heart rate | 0.98 (0.96, 1.01) | 0.31 | 1.02 (0.98, 1.06) | 0.46 | 0.95 (0.92, 0.99) | 0.02 |
| History of myocardial infarction | 0.50 (0.08, 3.05) | 0.45 | 1.29 (0.23, 7.26) | 0.77 | 0.37 (0.02, 5.74) | 0.48 |
| NT Pro-BNP (each 100 pg/mL increase) | 1.01 (1.00, 1.02) | 0.03 | 1.01 (1.00, 1.02) | 0.07 | 1.01 (0.99, 1.02) | 0.32 |
| hs-TNT (ng/mL)*1000 | 1.00 (0.99, 1.01) | 0.88 | 1.00 (0.99, 1.01) | 0.80 | 0.99 (0.94, 1.05) | 0.85 |
| **C-statistic (95% CI)** | **0.74 (0.65, 0.84)** |  | **0.80 (0.68, 0.91)** |  | **0.82 (0.71, 0.93)** |  |

Supplemental Table 14. Multivariable Cox proportional hazard risk models for the participants with LV ejection fraction ≥50% using original heart failure risk variables plus imaging and blood markers

|  | **First composite event** | | **Mortality** | | **Heart Failure Hospitalization** | |
| --- | --- | --- | --- | --- | --- | --- |
|  | **Adjusted HR (95% CI)** | **p-value** | **Adjusted HR (95% CI)** | **p-value** | **Adjusted HR (95% CI)** | **p-value** |
| Age | 1.01 (0.97, 1.05) | 0.61 | 1.01 (0.95, 1.06) | 0.82 | 1.03 (0.97, 1.10) | 0.35 |
| Male gender | 1.22 (0.51, 2.90) | 0.66 | 3.11 (0.86, 11.30) | 0.08 | 0.66 (0.17, 2.54) | 0.55 |
| Black | 1.79 (0.50, 6.43) | 0.38 | 1.06 (0.15, 7.64) | 0.96 | 3.93 (0.75, 20.66) | 0.11 |
| BMI | 1.00 (0.93, 1.06) | 0.91 | 0.95 (0.86, 1.06) | 0.37 | 1.01 (0.92, 1.11) | 0.81 |
| Smoking | 1.25 (0.53, 2.95) | 0.62 | 1.97 (0.64, 6.05) | 0.24 | 0.66 (0.16, 2.68) | 0.56 |
| History of hypertension | 2.06 (0.62, 6.82) | 0.24 | 3.58 (0.59, 21.93) | 0.17 | 1.21 (0.21, 6.78) | 0.83 |
| History of diabetes | 0.70 (0.23, 2.11) | 0.53 | 0.84 (0.19, 3.83) | 0.83 | 0.75 (0.15, 3.70) | 0.73 |
| Heart rate | 0.99 (0.96, 1.02) | 0.47 | 1.02 (0.97, 1.06) | 0.45 | 0.95 (0.91, 1.00) | 0.07 |
| History of myocardial infarction | 0.48 (0.08, 2.96) | 0.43 | 1.19 (0.20, 6.89) | 0.85 | 0.30 (0.02, 5.33) | 0.41 |
| Elevated ECV | 4.33 (1.79, 10.46) | 0.001 | 3.64 (1.09, 12.15) | 0.04 | 5.86 (1.59, 21.68) | 0.01 |
| Any scar presence | 1.44 (0.62, 3.33) | 0.40 | 1.20 (0.38, 3.79) | 0.76 | 2.03 (0.56, 7.42) | 0.28 |
| LVEF | 1.01 (0.97, 1.06) | 0.58 | 1.01 (0.95, 1.08) | 0.67 | 1.01 (0.94, 1.09) | 0.75 |
| LV mass index | 1.01 (1.00, 1.03) | 0.12 | 1.00 (0.97, 1.03) | 0.78 | 1.02 (1.00, 1.05) | 0.03 |
| NT Pro-BNP (each 100 pg/mL increase) | 1.01 (1.00, 1.02) | 0.04 | 1.01 (1.00, 1.02) | 0.08 | 1.01 (0.99, 1.03) | 0.19 |
| hs-TNT (ng/mL)*1000 | 1.00 (0.98, 1.01) | 0.85 | 1.00 (0.99, 1.01) | 0.86 | 0.96 (0.89, 1.04) | 0.34 |
| **C-statistic (95% CI)** | **0.78 (0.67, 0.89)** |  | **0.84 (0.72, 0.96)** |  | **0.85 (0.72, 0.98)** |  |

Supplemental Table 15. Multivariable Cox proportional hazard risk models for the participants with LV ejection fraction <50% using original heart failure risk variables

|  | **First composite event** | | **Mortality** | | **Heart Failure Hospitalization** | |
| --- | --- | --- | --- | --- | --- | --- |
|  | **Adjusted HR (95% CI)** | **p-value** | **Adjusted HR (95% CI)** | **p-value** | **Adjusted HR (95% CI)** | **p-value** |
| Age | 1.05 (1.01, 1.09) | 0.02 | 1.04 (0.97, 1.12) | 0.24 | 1.05 (1.00, 1.10) | 0.06 |
| Male gender | 0.73 (0.27, 1.95) | 0.53 | 0.63 (0.14, 2.84) | 0.55 | 0.53 (0.17, 1.66) | 0.27 |
| Black | 0.48 (0.15, 1.47) | 0.20 | 0.75 (0.14, 3.95) | 0.73 | 0.57 (0.15, 2.14) | 0.40 |
| BMI | 0.93 (0.87, 1.01) | 0.07 | 0.92 (0.80, 1.05) | 0.19 | 0.94 (0.87, 1.02) | 0.16 |
| Smoking | 0.67 (0.26, 1.70) | 0.40 | 0.92 (0.22, 3.79) | 0.91 | 0.66 (0.21, 2.10) | 0.49 |
| History of hypertension | 0.74 (0.21, 2.61) | 0.64 | NA | NA | 0.43 (0.11, 1.74) | 0.24 |
| History of diabetes | 2.47 (0.95, 6.42) | 0.06 | 2.07 (0.54, 7.85) | 0.29 | 2.92 (0.84, 10.16) | 0.09 |
| Heart rate | 1.04 (1.01, 1.07) | 0.003 | 1.06 (1.01, 1.11) | 0.01 | 1.03 (1.00, 1.07) | 0.046 |
| History of myocardial infarction | 0.83 (0.27, 2.51) | 0.74 | 1.30 (0.24, 6.97) | 0.76 | 0.58 (0.13, 2.48) | 0.46 |
| **C-statistic (95% CI)** | **0.73 (0.63, 0.83)** |  | **0.84 (0.76, 0.93)** |  | **0.73 (0.61, 0.85)** |  |

Supplemental Table 16. Multivariable Cox proportional hazard risk models for the participants with LV ejection fraction <50% using original heart failure risk variables plus imaging markers

|  | **First composite event** | | **Mortality** | | **Heart Failure Hospitalization** | |
| --- | --- | --- | --- | --- | --- | --- |
|  | **Adjusted HR (95% CI)** | **p-value** | **Adjusted HR (95% CI)** | **p-value** | **Adjusted HR (95% CI)** | **p-value** |
| Age | 1.05 (1.01, 1.10) | 0.02 | 1.07 (1.00, 1.16) | 0.06 | 1.06 (1.00, 1.12) | 0.07 |
| Male gender | 0.47 (0.15, 1.48) | 0.20 | 0.60 (0.09, 4.22) | 0.61 | 0.26 (0.07, 1.05) | 0.06 |
| Black | 0.47 (0.14, 1.51) | 0.20 | 0.71 (0.09, 5.55) | 0.74 | 0.52 (0.14, 1.99) | 0.34 |
| BMI | 0.94 (0.87, 1.01) | 0.11 | 0.96 (0.82, 1.11) | 0.57 | 0.94 (0.86, 1.02) | 0.15 |
| Smoking | 0.77 (0.28, 2.10) | 0.61 | 0.44 (0.07, 2.94) | 0.40 | 0.82 (0.26, 2.55) | 0.73 |
| History of hypertension | 0.76 (0.19, 3.06) | 0.70 | NA | NA | 0.45 (0.08, 2.49) | 0.36 |
| History of diabetes | 2.13 (0.77, 5.89) | 0.15 | 2.80 (0.51, 15.30) | 0.24 | 2.37 (0.63, 8.93) | 0.20 |
| Heart rate | 1.04 (1.01, 1.08) | 0.01 | 1.07 (1.01, 1.13) | 0.02 | 1.02 (0.99, 1.06) | 0.22 |
| History of myocardial infarction | 0.60 (0.16, 2.23) | 0.44 | 1.83 (0.23, 14.65) | 0.57 | 0.30 (0.06, 1.55) | 0.15 |
| Elevated ECV | 1.68 (0.66, 4.27) | 0.28 | 0.26 (0.05, 1.31) | 0.10 | 3.12 (0.92, 10.54) | 0.07 |
| Any scar presence | 1.02 (0.28, 3.70) | 0.98 | 1.06 (0.15, 7.64) | 0.96 | 1.58 (0.31, 8.05) | 0.58 |
| LVEF | 0.99 (0.95, 1.03) | 0.69 | 1.01 (0.93, 1.09) | 0.82 | 0.98 (0.93, 1.03) | 0.37 |
| LV mass index | 1.01 (0.99, 1.03) | 0.42 | 1.01 (0.97, 1.04) | 0.72 | 1.00 (0.98, 1.02) | 0.96 |
| **C-statistic (95% CI)** | **0.76 (0.66, 0.86)** |  | **0.85 (0.74, 0.96)** |  | **0.77 (0.66, 0.89)** |  |

Supplemental Table 17. Multivariable Cox proportional hazard risk models for the participants with LV ejection fraction <50% using original heart failure risk variables plus blood markers

|  | **First composite event** | | **Mortality** | | **Heart Failure Hospitalization** | |
| --- | --- | --- | --- | --- | --- | --- |
|  | **Adjusted HR (95% CI)** | **p-value** | **Adjusted HR (95% CI)** | **p-value** | **Adjusted HR (95% CI)** | **p-value** |
| Age | 1.05 (1.01, 1.09) | 0.02 | 1.05 (0.97, 1.12) | 0.23 | 1.04 (0.99, 1.09) | 0.09 |
| Male gender | 0.69 (0.26, 1.86) | 0.47 | 0.67 (0.13, 3.38) | 0.63 | 0.59 (0.18, 1.93) | 0.39 |
| Black | 0.40 (0.11, 1.47) | 0.17 | 0.77 (0.11, 5.47) | 0.79 | 0.31 (0.05, 1.74) | 0.18 |
| BMI | 0.94 (0.87, 1.01) | 0.11 | 0.92 (0.80, 1.05) | 0.21 | 0.95 (0.87, 1.03) | 0.23 |
| Smoking | 0.72 (0.29, 1.80) | 0.49 | 0.87 (0.21, 3.70) | 0.85 | 0.61 (0.19, 1.91) | 0.39 |
| History of hypertension | 0.57 (0.15, 2.10) | 0.39 | NA | NA | 0.29 (0.06, 1.35) | 0.12 |
| History of diabetes | 2.57 (0.93, 7.10) | 0.07 | 2.07 (0.54, 7.91) | 0.29 | 3.33 (0.88, 12.65) | 0.08 |
| Heart rate | 1.03 (1.00, 1.06) | 0.03 | 1.06 (1.01, 1.12) | 0.03 | 1.02 (0.98, 1.05) | 0.39 |
| History of myocardial infarction | 0.61 (0.20, 1.91) | 0.40 | 1.34 (0.24, 7.62) | 0.74 | 0.42 (0.10, 1.88) | 0.26 |
| NT Pro-BNP (each 100 pg/mL increase) | 1.01 (1.00, 1.01) | 0.24 | 1.00 (0.99, 1.01) | 1.00 | 1.01 (1.00, 1.02) | 0.04 |
| hs-TNT (ng/mL)*1000 | 1.00 (1.00, 1.01) | 0.01 | 1.00 (1.00, 1.01) | 0.74 | 1.00 (1.00, 1.01) | 0.046 |
| **C-statistic (95% CI)** | **0.75 (0.64, 0.86)** |  | **0.84 (0.75, 0.93)** |  | **0.75 (0.63, 0.88)** |  |

Supplemental Table 18. Multivariable Cox proportional hazard risk models for the participants with LV ejection fraction <50% using original heart failure risk variables plus imaging and blood markers

|  | **First composite event** | | **Mortality** | | **Heart Failure Hospitalization** | |
| --- | --- | --- | --- | --- | --- | --- |
|  | **Adjusted HR (95% CI)** | **p-value** | **Adjusted HR (95% CI)** | **p-value** | **Adjusted HR (95% CI)** | **p-value** |
| Age | 1.05 (1.00, 1.10) | 0.04 | 1.09 (1.00, 1.19) | 0.053 | 1.04 (0.98, 1.11) | 0.18 |
| Male gender | 0.69 (0.21, 2.33) | 0.55 | 0.89 (0.10, 7.71) | 0.91 | 0.39 (0.09, 1.80) | 0.23 |
| Black | 0.33 (0.08, 1.31) | 0.12 | 1.07 (0.11, 10.21) | 0.96 | 0.24 (0.04, 1.45) | 0.12 |
| BMI | 0.95 (0.87, 1.02) | 0.17 | 0.99 (0.84, 1.16) | 0.87 | 0.95 (0.86, 1.05) | 0.29 |
| Smoking | 0.71 (0.26, 1.96) | 0.51 | 0.31 (0.04, 2.20) | 0.24 | 0.60 (0.18, 2.07) | 0.42 |
| History of hypertension | 0.57 (0.13, 2.40) | 0.44 | NA | NA | 0.35 (0.06, 2.08) | 0.25 |
| History of diabetes | 2.26 (0.78, 6.56) | 0.13 | 4.03 (0.56, 29.28) | 0.17 | 2.84 (0.72, 11.22) | 0.14 |
| Heart rate | 1.03 (1.00, 1.06) | 0.06 | 1.07 (1.00, 1.14) | 0.04 | 1.01 (0.97, 1.05) | 0.68 |
| History of myocardial infarction | 0.52 (0.14, 1.87) | 0.32 | 1.69 (0.21, 13.55) | 0.62 | 0.26 (0.05, 1.40) | 0.12 |
| Elevated ECV | 1.90 (0.77, 4.68) | 0.16 | 0.20 (0.03, 1.17) | 0.07 | 3.54 (1.04, 12.08) | 0.04 |
| Any scar presence | 0.90 (0.22, 3.61) | 0.88 | 0.81 (0.10, 6.46) | 0.85 | 2.06 (0.31, 13.62) | 0.45 |
| LVEF | 1.01 (0.97, 1.06) | 0.59 | 1.04 (0.93, 1.16) | 0.50 | 1.01 (0.95, 1.06) | 0.82 |
| LV mass index | 1.00 (0.98, 1.02) | 0.98 | 0.99 (0.95, 1.03) | 0.67 | 0.99 (0.97, 1.02) | 0.68 |
| NT Pro-BNP (each 100 pg/mL increase) | 1.01 (1.00, 1.02) | 0.14 | 1.00 (0.99, 1.01) | 0.93 | 1.01 (1.00, 1.03) | 0.02 |
| hs-TNT (ng/mL)*1000 | 1.01 (1.00, 1.01) | 0.02 | 1.01 (1.00, 1.02) | 0.23 | 1.00 (1.00, 1.01) | 0.12 |
| **C-statistic (95% CI)** | **0.78 (0.67, 0.88)** |  | **0.84 (0.71, 0.97)** |  | **0.79 (0.67, 0.91)** |  |
